# A novel approach to identifying patterns of human invasion-inhibitory antibodies guides the design of malaria vaccines incorporating polymorphic antigens

**DOI:** 10.1186/s12916-016-0691-6

**Published:** 2016-09-23

**Authors:** Damien R. Drew, Danny W. Wilson, Salenna R. Elliott, Nadia Cross, Ulrich Terheggen, Anthony N. Hodder, Peter M. Siba, Kiprotich Chelimo, Arlene E. Dent, James W. Kazura, Ivo Mueller, James G. Beeson

**Affiliations:** 1 The Burnet Institute of Medical Research and Public Health, 85 Commercial Road, Melbourne, Victoria 3004 Australia; 2 Research Centre for Infectious Diseases, School of Molecular and Biomedical Science, University of Adelaide, Adelaide, South Australia Australia; 3 Department of Medicine, Royal Melbourne Hospital, University of Melbourne, Parkville, Victoria Australia; 4 Papua New Guinea Institute of Medical Research, Goroka, Papua New Guinea; 5 Kenya Medical Research Institute, Kisian, Kenya; 6 Case Western Reserve University, Cleveland, Ohio USA; 7 Walter and Eliza Hall Institute of Medical Research, Parkville, Victoria Australia; 8 Department of Microbiology, Monash University, Clayton, Victoria Australia

**Keywords:** *Plasmodium falciparum*, Malaria, Vaccines, Apical membrane antigen 1, Antibodies, Immunity

## Abstract

**Background:**

The polymorphic nature of many malaria vaccine candidates presents major challenges to achieving highly efficacious vaccines. Presently, there is very little knowledge on the prevalence and patterns of functional immune responses to polymorphic vaccine candidates in populations to guide vaccine design. A leading polymorphic vaccine candidate against blood-stage *Plasmodium falciparum* is apical membrane antigen 1 (AMA1), which is essential for erythrocyte invasion. The importance of AMA1 as a target of acquired human inhibitory antibodies, their allele specificity and prevalence in populations is unknown, but crucial for vaccine design.

**Methods:**

*P. falciparum* lines expressing different AMA1 alleles were genetically engineered and used to quantify functional antibodies from two malaria-exposed populations of adults and children. The acquisition of AMA1 antibodies was also detected using enzyme-linked immunosorbent assay (ELISA) and competition ELISA (using different AMA1 alleles) from the same populations.

**Results:**

We found that AMA1 was a major target of naturally acquired invasion-inhibitory antibodies that were highly prevalent in malaria-endemic populations and showed a high degree of allele specificity. Significantly, the prevalence of inhibitory antibodies to different alleles varied substantially within populations and between geographic locations. Inhibitory antibodies to three specific alleles were highly prevalent (FVO and W2mef in Papua New Guinea; FVO and XIE in Kenya), identifying them for potential vaccine inclusion. Measurement of antibodies by standard or competition ELISA was not strongly predictive of allele-specific inhibitory antibodies. The patterns of allele-specific functional antibody responses detected with our novel assays may indicate that acquired immunity is elicited towards serotypes that are prevalent in each geographic location.

**Conclusions:**

These findings provide new insights into the nature and acquisition of functional immunity to a polymorphic vaccine candidate and strategies to quantify functional immunity in populations to guide rational vaccine design.

**Electronic supplementary material:**

The online version of this article (doi:10.1186/s12916-016-0691-6) contains supplementary material, which is available to authorized users.

## Background

The development of effective vaccines is a major global goal towards achieving malaria control and elimination. However, a major challenge in the development of highly efficacious vaccines is antigen polymorphism, which is an important issue for many leading vaccine candidates. This includes the RTS,S vaccine, which recently completed phase III trials, and vaccines based on apical membrane antigen 1 (AMA1) and merozoite surface protein 2 (MSP2); for all these candidates, vaccine efficacy against malaria or *Plasmodium falciparum* infection was higher for episodes caused by vaccine-like strains compared to vaccine-dissimilar strains [1–3]. Currently, knowledge on the distribution and prevalence in populations of functional immune responses to different alleles or strains for polymorphic vaccine candidates is very limited, but would be highly valuable for guiding vaccine design. A further constraint to vaccine development is a paucity of data on the targets of functional immune responses that may mediate protective immunity. Antibodies form an important component of acquired human immunity [4–6]. Merozoite antigens are important targets of antibodies that inhibit erythrocyte invasion, limit parasite replication and control disease associated with blood stage replication [5, 7]. However, the major targets of acquired invasion-inhibitory antibodies are unclear.

The merozoite protein apical membrane antigen 1 (AMA1) is a leading polymorphic vaccine candidate that plays an essential role in host cell invasion and is a prominent target of naturally acquired antibodies [7–9]. AMA1 binds the rhoptry neck protein, RON2, a key interaction that is required for formation of the tight junction during invasion [10, 11], and antibodies to AMA1 inhibit invasion in vitro [12–18]. In malaria-exposed individuals, antibodies to AMA1 are highly prevalent, increasing with age and exposure [19–22], and some studies have found antibodies to AMA1, measured by standard enzyme-linked immunosorbent assay (ELISA), are associated with protection from malaria in longitudinal studies [7, 8, 20, 23–27]. Affinity-purified human antibodies to AMA1 can inhibit invasion [12], and some people acquire antibodies to inhibitory epitopes of AMA1 [28]. However, the significance of AMA1 as a target of acquired invasion-inhibitory antibodies and the strain specificity and prevalence of these antibodies remain unknown [29].

AMA1 is highly polymorphic with more than 200 haplotypes, and reflects the challenges faced in vaccine development of overcoming antigenic diversity to enable highly efficacious vaccines [12–15, 30–32]. Humans generate both allele-specific and cross-reactive antibodies to AMA1 [20, 21, 32], but how these antibodies are acquired and their relative contribution to protection remain uncertain, particularly for functional antibodies. A phase II trial in Malian children of an AMA1 vaccine containing a single allele demonstrated significant strain-specific efficacy, reducing the risk of malaria caused by vaccine-like strains (defined by genotype) [2]. These results provide an important proof of concept for AMA1-based vaccines, but highlight the need to understand AMA1 antigenic diversity and address this diversity in vaccine design. Although there are more than 200 AMA1 haplotypes, antigenic diversity appears more limited than suggested by sequence diversity [31, 32]. Population genetics suggest that the distribution of AMA1 haplotypes, or major haplotype groupings, is similar across different geographic regions and that there is a similar proportion of major haplotype clusters within a population [33, 34]. However, data on the acquisition of functional antibodies in populations is absent due to a lack of tools to measure these antibodies. Understanding these issues is crucial for vaccine design with respect to selecting alleles for inclusion in future vaccines and determining whether vaccine formulations may need to differ between regions. Similar needs exist for many other vaccine candidates for which antigenic diversity is a key issue [1, 35].

To date, there has been no way of quantifying naturally acquired AMA1-specific invasion-inhibitory antibodies in human populations or quantifying the prevalence of allele-specific functional antibodies. Moreover, the prevalence of allele-specific functional antibodies has not been reported for any malaria antigen to date, because of a lack of tools and approaches. In this study we used AMA1 as a model to investigate the patterns, prevalence and nature of acquired functional immunity to a polymorphic vaccine candidate. We developed a novel approach using genetically engineered *P. falciparum* lines expressing one of six antigenically distinct AMA1 alleles that broadly represent global antigenic diversity [15, 31]. The development of these novel tools enabled us to evaluate the importance of AMA1 as a target of human invasion-inhibitory antibodies, determine the extent to which inhibitory antibodies are cross-reactive and allele-specific and determine whether the prevalence of inhibitory antibodies varies for different allelic serotypes. This knowledge will be highly valuable in selecting alleles for potential vaccine inclusion and advancing our understanding of strain-specific protection, which is a feature of immunity to malaria and relevant to many malaria vaccine candidates.

## Methods

### Aim

The aim of this study was to measure and compare the prevalence and allele specificity of naturally acquired growth inhibitory antibodies to a polymorphic malaria antigen (AMA1) across two geographically distinct locations (Kenya and Papua New Guinea) to inform the design of future vaccines incorporating polymorphic antigens.

### Design

*P. falciparum* lines were genetically engineered to express one of six antigenically distinct alleles of AMA1 and were used in in vitro growth inhibition assays (GIAs) to measure the presence of AMA allele-specific functional antibodies in samples from children and adults in Papua New Guinea and adults living in Kenya. Antibodies to AMA1 were also quantified by standard ELISA and competition ELISA. 

### Study population and setting

Blood was collected (in 2004) from adults and children participating in a cross-sectional study in Madang Province, Papua New Guinea (PNG), a region that experiences year-round malaria transmission with seasonal variation [32, 36]. Sera were collected from 50 adults (median age 28 years (interquartile range, IQR 24.8–35)) and 49 children (median age 7 years (IQR 6–9)), with 53 % male and 48.2 % being positive for parasitemia (18 % *P. falciparum*; 26 % *P. vivax*; 4 % *P. malariae*; 5 % mixed infections). Serum samples were also collected from adult residents (*n* = 54) of Nyanza Province, Kenya, an area of high malaria transmission [37]. Sera from non-immune Melbourne donors (provided by the Australian Red Cross Blood Service) were used as negative controls. Ethical approval was obtained from the Medical Research Advisory Committee, PNG, Kenya Medical Research Institute, Walter and Eliza Hall Institute and Alfred Health Human Research Ethics Committees, Australia.

### Antibodies, immunoblots and ELISA

Rabbit antisera against recombinant, refolded AMA1 ectodomains were generated and purified as described [12, 31]. Sodium dodecyl sulphate-polyacrylamide gel electrophoresis (SDS-PAGE) and immunoblot analysis of parasite proteins was performed as previously described [31]. Standard ELISAs to measure AMA1 antibodies in plasma and competition ELISAs with different AMA1 alleles were also performed as described [8, 32]. See Additional file 1 for more details.

### Parasite lines, culture and invasion inhibition assays

Wild-type W2Mef *P. falciparum* and transgenic W2Mef *P. falciparum* genetically engineered to express one of six different AMA1 alleles were cultured in vitro [31]. Transgenic lines expressing W2Mef, 3D7 and FVO AMA1 alleles were generated in a previous study [31], while lines expressing HB3, XIE and Pf2006 AMA1 alleles were generated in this study using previously described techniques [31]. Flow cytometry-based invasion inhibition assays were performed as described in detail elsewhere [6, 31, 38, 39]. In brief, synchronised *P. falciparum* (pigmented trophozoite stage) from culture was adjusted to 0.1 % parasitemia and 2 % haematocrit for assay set-up. Human serum samples were tested for growth inhibition at 1/10 dilution in duplicate or triplicate wells in a 96-well format. Parasites were allowed to develop through two cycles of erythrocyte invasion for 72 hours at 37 °C. Parasitemia was evaluated by flow cytometry (see Additional file 1 for further details). All PNG samples were tested in duplicate in at least two independent assays; the means of all results (duplicate wells and assays) were used in analysis. Subsequently, a subset of 66 samples was tested in duplicate in a repeat assay against all six parasite lines in parallel to further validate and confirm the findings obtained. After establishing the approach and reproducibility with the PNG samples, all Kenyan samples were tested together against all six AMA1 transgenic lines in duplicate in a single assay, and the mean of the results for each sample was used for analysis. Samples from malaria-naïve Australian residents were included as non-immune controls in all assays. AMA1-specific invasion inhibition was considered to be present in a sample if the sample inhibited one of the genetically engineered *P. falciparum* lines compared with other lines tested at the same time in parallel by at least 10 % or higher greater (defined as the absolute difference of 10 % or higher, not a relative difference of 10 %).This cut-off was selected on the following basis. (1) Variation in the assay was estimated as +/-2 standard deviations around the mean of the Melbourne control sera used in assays; 2 standard deviations was used to define our ‘variation threshold’, and this value was always less than 10 % across assays, as we have found in previous studies [39]. Assay variance was similar when using samples from our study populations (examples are shown in Fig. 3 and will be discussed below in the Results section). (2) It was considered that an absolute difference of 10 % was currently regarded as an acceptable difference when reporting results from inhibition assays. To assess assay variation, we calculated the coefficient of variation (which represents standard deviation relative to the mean; CV). The CV values for the sample sets tested were as follows: 2.2–4.6 % for individual malaria non-exposed control samples; 3.2–4 % for PNG samples and 3.6–6.6 % for Kenyan samples (the range of CV values shown represents values calculated across different assays and using different AMA1 transgenic lines). Detailed evaluations of the reproducibility and precision of these assays and their application in measuring antigen-specific inhibitory antibodies have been published elsewhere [6, 38–41].

### Statistical analysis

Analyses were performed using GraphPad Prism 5. Invasion inhibition of different *P. falciparum* lines was compared by paired *t* test, or Wilcoxon’s matched-pairs signed-rank sum test for non-parametric data. Prevalence of AMA1 antibodies or prevalence of specific invasion inhibition was compared using the chi-squared test or Fisher’s exact test. The Mann-Whitney test was used to compare antibody levels. Spearman’s correlation coefficient was used to assess associations between antibodies to different AMA1 alleles measured by ELISA, and between invasion inhibition and AMA1 antibodies.

## Results

### Construction and phenotyping of genetically engineered *P. falciparum* lines

**Fig. 1:**
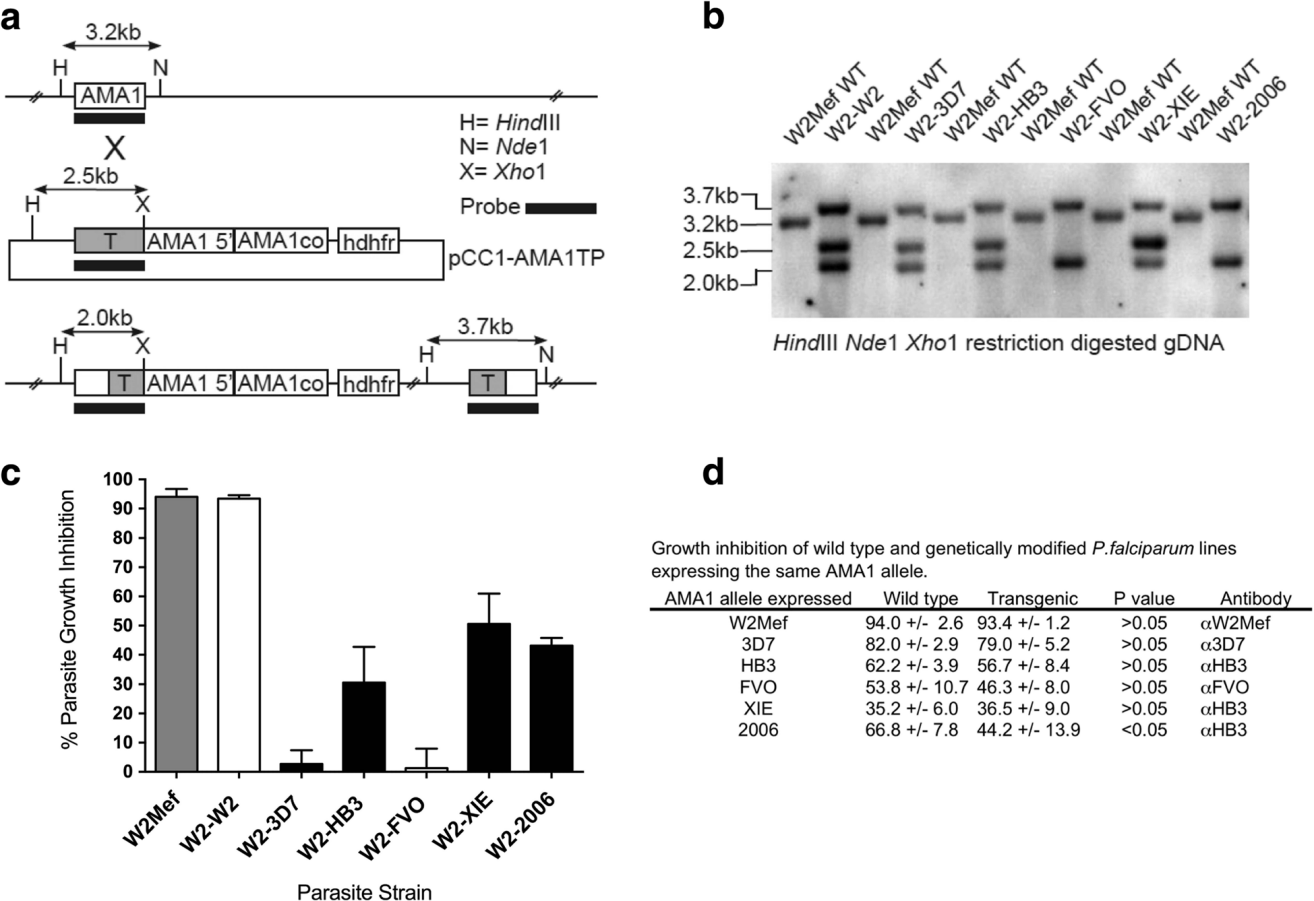
Generation and phenotypic analysis of *P. falciparum* lines genetically engineered to express different AMA1 alleles. **a** Plasmid design and integration: HB3, XIE and Pf2006 codon-optimised AMA1 alleles were transfected into W2Mef parental parasites. The single crossover event for allelic replacement of the W2Mef wild-type (*WT*) AMA1 with the codon-optimised AMA1 alleles (AMA1co) is illustrated. **b** Southern blots: genomic DNA from parental W2Mef (W2Mef WT) and genetically engineered parasite lines (W2-W2, W2-3D7, W2-FVO, W2-HB3, W2-XIE and W2-2006) were digested with restriction enzymes as indicated. Expected sizes for WT, non-integrated plasmid and for integrated codon-optimised AMA1 alleles are shown in kilobases (*kb*). **c** Differential invasion inhibition of W2Mef and genetically engineered parasite lines in the presence of W2Mef AMA1 rabbit anti-serum (1:10 dilution) in a two-cycle growth inhibition assay (*GIA*). Columns represent the mean percentage growth inhibition (relative to a non-inhibitory control) achieved in two separate assays tested in triplicate wells. Antibody inhibition was significantly lower for W2-3D7, W2-HB3, W2-FVO, W2-2006 and W2-XIE than for W2mef parental or W2-W2 (*P* < 0.05 by paired *t* test). **d** Antibody-mediated invasion inhibition of wild-type or genetically engineered *P. falciparum* expressing the same AMA1 allele was compared in GIAs. Total inhibition is expressed as the mean of two separate assays performed in triplicate. *n* = 6 for each group listed. Error bars indicate + standard error of the mean (*SEM*). Statistical analysis was by paired *t* test

We generated *P. falciparum* isolates from a common parental line (W2Mef) which were genetically engineered to express one of six different AMA1 alleles that broadly represent the antigenic diversity of AMA1, including alleles from our study population [31, 32]. We have previously described the generation of W2Mef parasite lines that express W2Mef, 3D7 or FVO AMA1 alleles (W2-W2, W2-3D7 and W2-FVO, respectively [31]). Here, we expanded our repertoire of genetically engineered lines to include W2Mef parasites that express HB3, XIE or Pf2006 AMA1 alleles (W2-HB3, W2-XIE and W2-2006, respectively). We constructed plasmids containing codon-optimised HB3, XIE and Pf2006 AMA1 alleles (Fig. 1a). These plasmids were transfected into W2Mef parental parasites and successfully integrated into the wild-type (WT) AMA1 locus following drug selection (Fig. 1b). In invasion inhibition assays using W2Mef AMA1 antibodies (generated in rabbits), no significant difference in growth inhibition was detected between W2Mef parental and the genetically engineered W2-W2 parasites used as a transgenic control line (Fig. 1c), confirming that both strains express equivalent amounts of W2Mef AMA1. All other genetically engineered strains were significantly less inhibited by W2Mef AMA1 anti-serum than the W2Mef parental strain, confirming the loss of endogenous W2Mef AMA1 expression (paired *t* test *P* < 0.05, Fig. 1c). Whilst W2-3D7 and W2-FVO parasites effectively escaped W2Mef AMA1 antibody inhibition, W2-HB3, W2-XIE and W2-2006 genetically engineered parasites were still inhibited to some degree, consistent with known cross-strain AMA1 antibody inhibition [16, 31].

The correct expression of AMA1 alleles in genetically engineered *P. facliparum* lines was also confirmed by Western blot (Additional file 1: Figure S1) and inhibition assays with WT parasite strains (Fig. 1d). *P. falciparum* expressing W2mef, 3D7 and FVO alleles have previously been shown to express the correct allele of AMA1 [31], and this was also shown for W2-HB3 parasites (Additional file 1: Figure S1A). As we did not have antibodies raised against the XIE or Pf2006 AMA1 alleles, expression of these alleles in W2-XIE and W2-2006 parasites was confirmed by probing Western blots with HB3 AMA1 antibodies, which cross-reacted with these alleles sufficiently strongly for detection in Western blots, but not with W2Mef AMA1 (Additional file 1: Figure S1B, C). Parental and genetically engineered parasite lines expressing the same AMA1 alleles showed equivalent levels of invasion inhibition with allele-specific antibodies (Fig. 1d), and 3D7-WT and W2-3D7 lines were equally inhibited by the AMA1-binding protein R1 (data not shown), further demonstrating that the level of AMA1 expressed in the parental and transfected lines was similar.

### Human AMA1 antibodies measured by ELISA

**Fig. 2:**
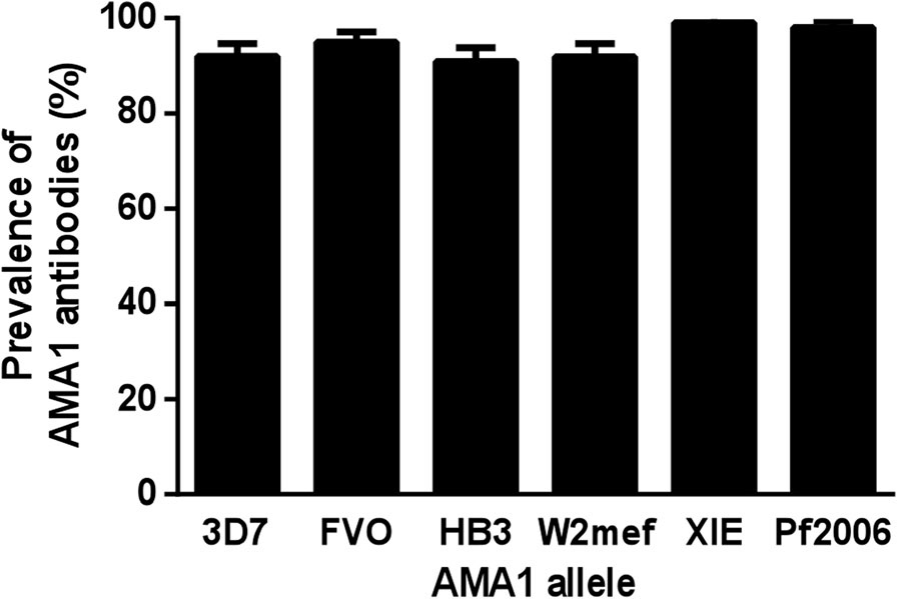
Prevalence of antibodies to different AMA1 alleles measured by ELISA. Error bars indicate + standard error. (*n* = 98 or 99 samples tested for each allele)

We first measured antibodies to recombinant AMA1 in the PNG study population by standard ELISA. The prevalence of AMA1 antibodies was high (>90 %) and similar for each of the six AMA1 alleles that were expressed by the genetically engineered *P. falciparum* lines (chi-square *P* = 0.07). See Fig. 2; (relative antibody levels are shown in Additional file 1: Figure S2). Antibody responses were strongly correlated between alleles (Spearman’s ρ = 0.74–0.97, *P* < 0.0001).

### Allele-specific AMA1 invasion-inhibitory antibodies

**Fig. 3:**
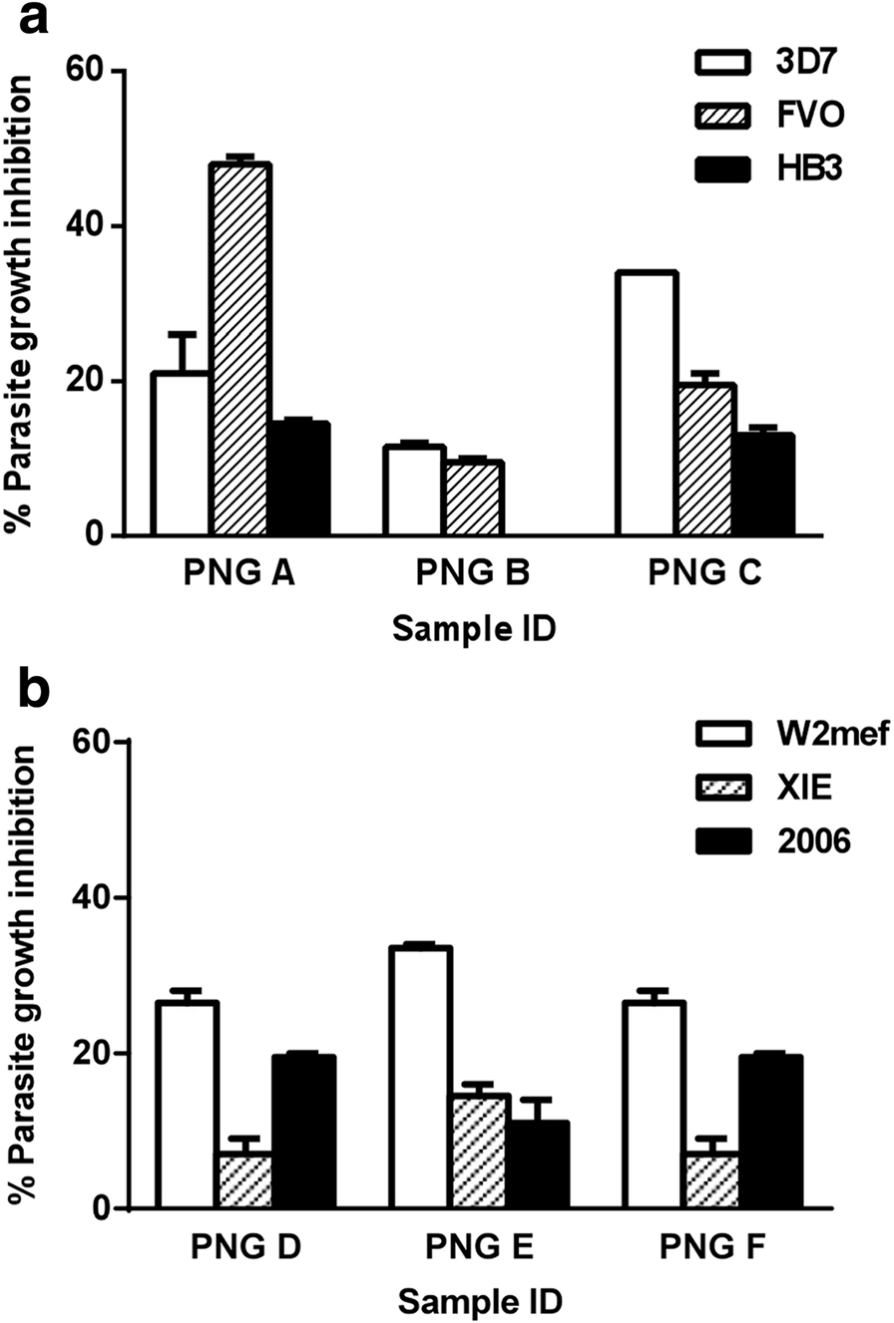
Representative examples of differential growth inhibition of genetically engineered *P. falciparum* by human antibodies in the PNG study . Comparisons are shown of lines tested in parallel (3D7, FVO and HB3 (**a**) or W2mef, XIE and Pf2006 (**b**)) for inhibition by serum samples from malaria-exposed PNG individuals. Columns represent mean percentage growth inhibition (relative to control) of duplicate wells from a single experiment. Error bars indicate + SEM

**Fig. 4:**
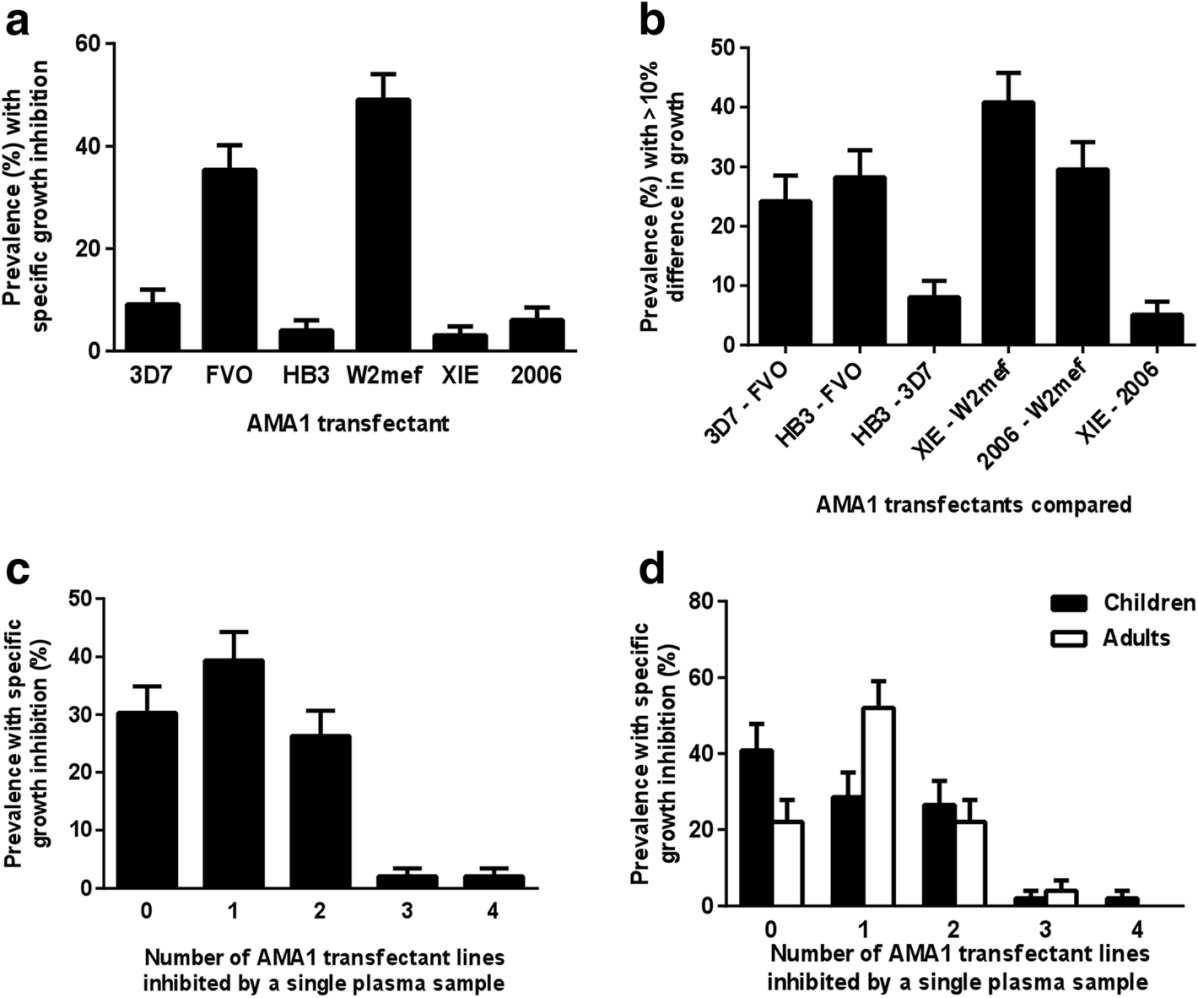
Differential invasion inhibition of genetically engineered *P. falciparum* expressing different AMA1 alleles by antibodies in the PNG study. **a** Prevalence of specific invasion inhibition (at least 10 % greater inhibition of one *P. falciparum* line compared with either of two other lines tested at the same time) amongst PNG serum samples (*n* = 98 or 99). **b** Prevalence of PNG serum samples showing difference in invasion inhibition >10 % between two *P. falciparum* lines (line A – line B) tested simultaneously (*n* = 98 or 99). Comparisons were made between lines tested together (3D7, FVO and HB3, or W2mef, XIE and Pf2006). Error bars indicate + standard error (comparison of 3D7-FVO and HB3-FVO versus HB3-3D7, *P* = 0.003 and *P* = 0.0004, respectively; XIE-W2Mef or Pf2006-W2Mef versus XIE-Pf2006, *P* < 0.0001). **c**, **d** Breadth of AMA1-specific growth-inhibitory activity in PNG population: **c** overall (*n* = 98 or 99) and **d** amongst children (*n* = 48 or 49) versus adults (*n* = 50). Specific growth inhibition was considered present when there was at least 10 % greater inhibition (absolute difference) of one genetically engineered *P. falciparum* line compared with either of two other lines tested at the same time (3D7, FVO and HB3, or W2mef, XIE and Pf2006). There was a significant difference between children and adults in the proportion of samples that differentially inhibited only one isolate (*P* < 0.05, Fisher’s exact test). Error bars indicate + standard error

Differential invasion inhibition of AMA1 genetically engineered *P. falciparum* lines by serum antibodies from malaria-exposed individuals provided evidence that AMA1 is a target of naturally acquired invasion-inhibitory antibodies (Fig. 3a and b). Invasion inhibition was considered specific if there was at least 10 % or greater inhibition (absolute difference) of one genetically engineered *P. falciparum* line compared with either of two other lines tested at the same time in parallel (Fig. 4a). It was striking to see a substantially higher prevalence of inhibitory antibodies for FVO and W2mef compared to other alleles (Fisher’s exact test, *P* < 0.0001 (Fig. 4a)), despite there being a similar prevalence of total immunoglobulin G (IgG) to each allele when measured by standard ELISA; levels of antibodies determined by ELISA were not clearly predictive of the pattern of inhibitory antibodies (Additional file 1: Figure S2). The prevalence of specific invasion inhibition for each AMA1 allele was not markedly different between adults and children (*P* = 0.09) (Additional file 1: Figure S3A). Pair-wise comparisons between different AMA1 lines for inhibition by serum antibodies further demonstrated that the prevalence of invasion-inhibitory samples was substantially higher for FVO and W2mef than for comparison alleles (Fig. 4b). Furthermore, the levels of inhibitory antibodies were higher for FVO and W2mef than for the comparison lines. For example, inhibition of the FVO expressing line was significantly greater than 3D7 (*P* < 0.0001; Wilcoxon’s matched-pairs signed-rank sum test), and inhibition of W2mef was significantly greater than XIE or Pf2006 (*P* < 0.01 and *P* < 0.05, respectively). There was no significant difference in prevalence between children and adults for any comparison (Fisher’s exact test, *P* > 0.12) (Additional file 1: Figure S3B). These findings clearly indicate that the prevalence and levels of inhibitory antibodies to different AMA1 alleles vary significantly within a population, which was not clearly evident from standard ELISA approaches, and has significant implications for vaccine design.

### Specificity and relatedness of invasion-inhibitory responses

Analysis of the specificity or cross-reactivity of inhibitory antibodies revealed that most serum samples were not broadly cross-inhibitory with few samples inhibiting more than two different AMA1 lines (Fig. 4c, Additional file 1: Table S1). Children and adults showed some differences in the profile of allele-specific inhibitory activity (Fig. 4d), with a higher proportion of children showing no specific inhibition of any genetically engineered *P. falciparum* line (Fisher’s exact test *P* = 0.053), and a greater proportion of adults had specific inhibitory activity against one genetically engineered *P. falciparum* line (*P* = 0.024).

**Fig. 5:**
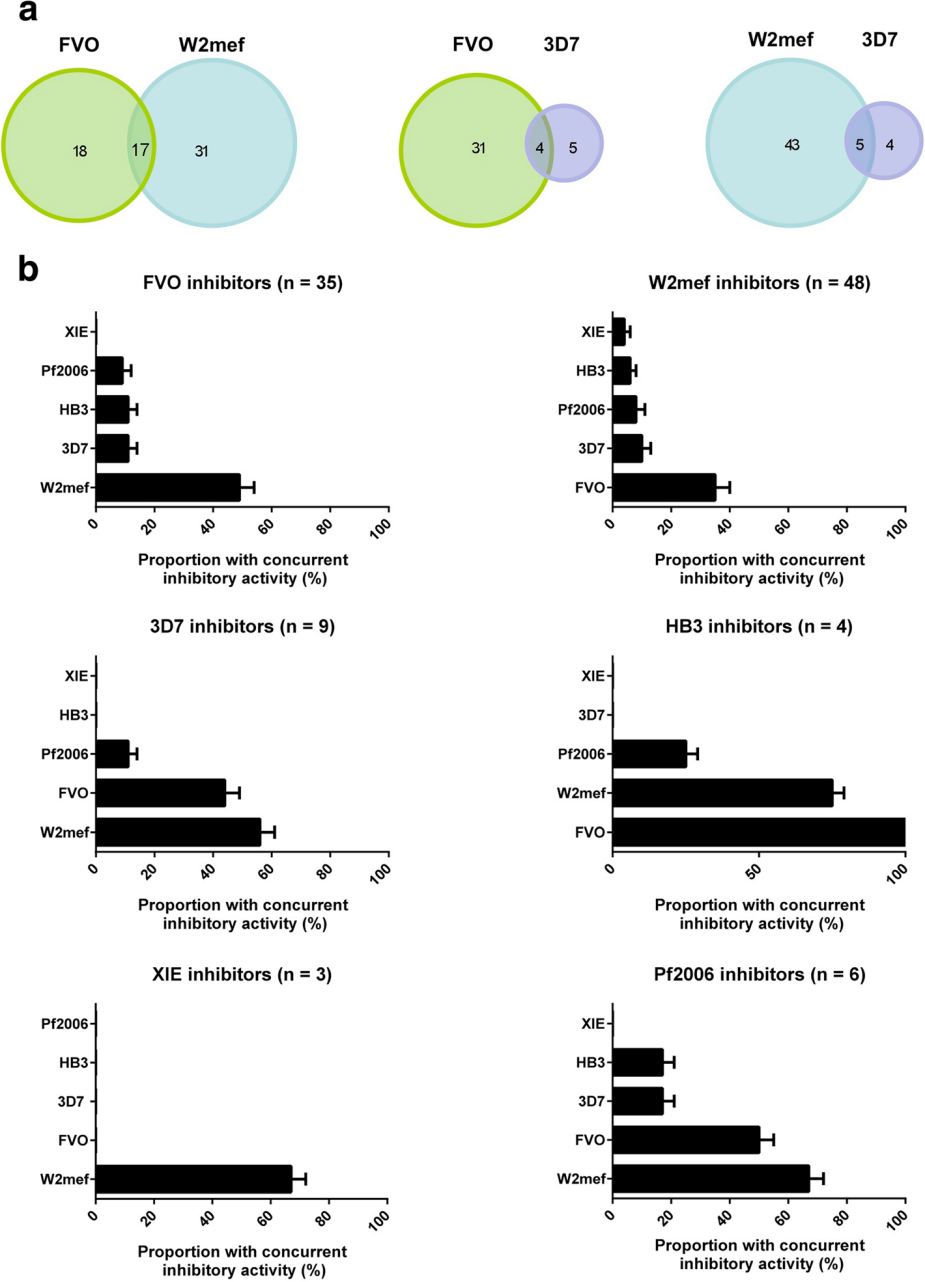
Overlap and allele specificity of AMA1-specific invasion-inhibitory antibodies. **a** Overlap in AMA1-specific invasion-inhibitory activity for the three AMA1 alleles with the highest prevalence of inhibitory antibodies. Numbers of PNG individuals whose serum showed specific inhibition of each line are shown. **b** Proportion of PNG samples (*n* = 98 or 99) showing specific inhibition of genetically engineered *P. falciparum* expressing one allele of AMA1 that also inhibited growth of lines expressing other AMA1 alleles. Error bars indicate + SEM

The pattern of overlap suggested individuals had predominantly acquired mixtures of allele-specific invasion-inhibitory antibodies of multiple specificities, rather than truly cross-inhibitory antibodies. Overlap in inhibitory activity was related to the prevalence of specific inhibitory antibodies targeting each AMA1 allele. A sample with inhibitory activity specific for any AMA1 allele was most likely to show concurrent inhibitory activity against FVO and/or W2mef (Fig. 5a, Additional file 1: Table S1), because the prevalence of inhibitory antibodies to these AMA1 alleles was highest. Whilst a proportion of samples inhibited both FVO and W2mef lines (49 % of FVO inhibitors also inhibited W2mef and 35 % of W2mef inhibitors also inhibited FVO) (Fig. 5b, Additional file 1: Table S1), a considerable number of samples only inhibited one line or the other, consistent with these antibodies being allele-specific rather than cross-inhibitory. When samples that showed overlap in invasion-inhibitory activity were compared with those that inhibited only a single genetically engineered *P. falciparum* line (e.g. FVO and W2mef versus FVO only), no difference was observed in antibody level by ELISA (*P* > 0.347, Mann-Whitney U test) or absolute invasion inhibition (*P* > 0.7011) (Additional file 1: Figure S4). This suggests that overlap in invasion-inhibitory activity is not related to antibody magnitude.

### Invasion inhibition versus AMA1 antibodies measured by standard ELISA

**Fig. 6:**
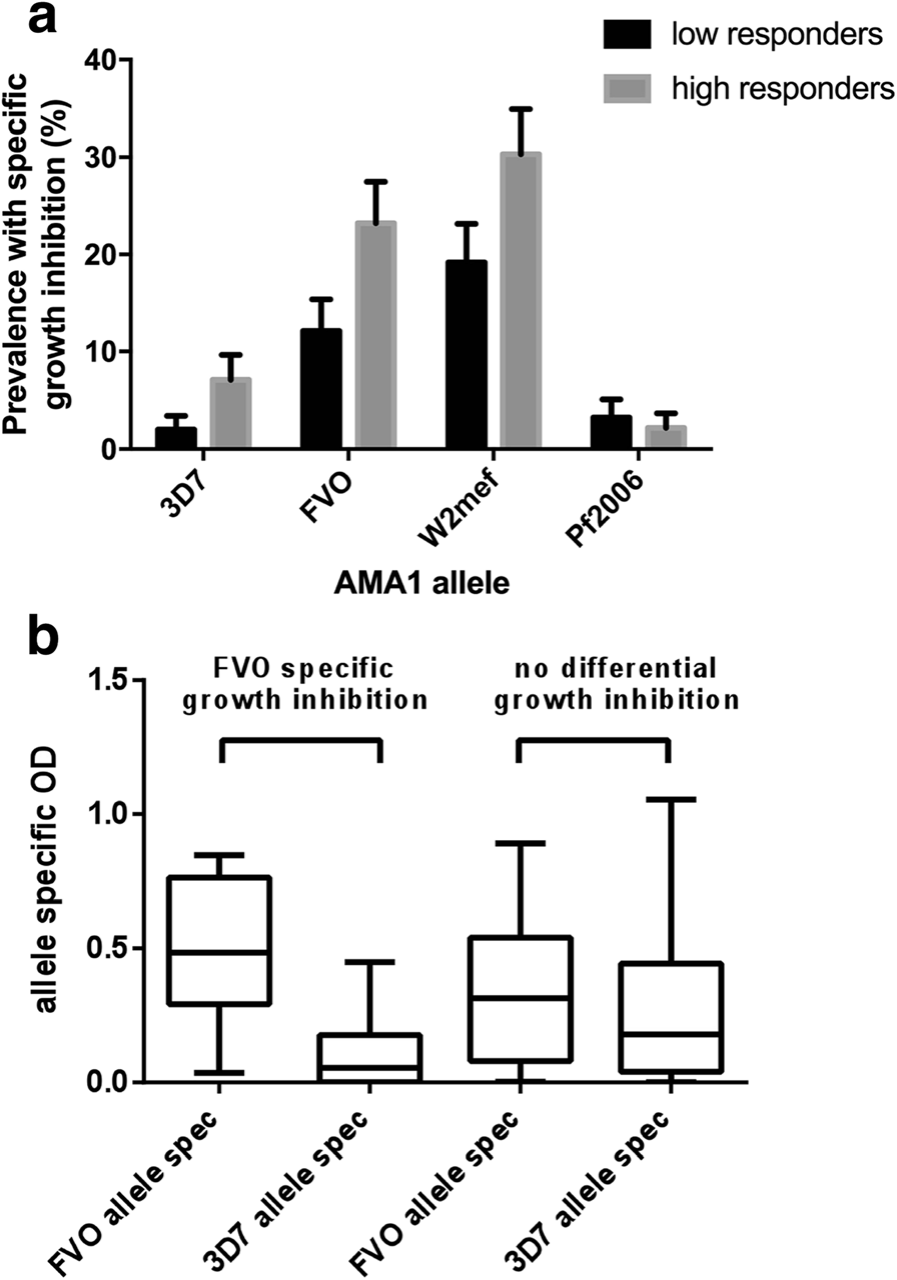
AMA1-specific growth inhibition in low and high AMA1 antibody responders determined by ELISA. **a** Prevalence of specific growth inhibition of genetically engineered *P. falciparum* expressing different AMA1 alleles (>10 % inhibition of one line compared with either of two other lines tested simultaneously), according to level of antibody response to specific AMA1 alleles determined by ELISA (high responses classified as greater than the median value) (*n* = 99). Prevalence of specific inhibition for HB3 and XIE was too low to be included in the analysis. Error bars indicate standard error. There was a significant difference in the prevalence of FVO-specific and W2mef-specific inhibitory antibodies between low and high responders by ELISA (*P* < 0.05, Fisher’s exact test). **b** Allele-specific antibodies to FVO and 3D7 AMA1 were measured by competition ELISA in selected serum samples that showed FVO-specific growth inhibition (growth of genetically engineered *P. falciparum* expressing FVO at least 10 % lower than growth of genetically engineered *P. falciparum* expressing 3D7 AMA1 (*n* = 24)), and amongst samples with no differential growth inhibition (*n* = 64). Higher levels of FVO allele-specific antibodies (FVO AMA1-specific antibodies that do not cross-react with 3D7 AMA) than 3D7 allele-specific antibodies (3D7 AMA1-specific antibodies that do not cross-react with FVO AMA1) were observed amongst samples with FVO-specific growth inhibition (Mann-Whitney U test, *P* < 0.0001)

Our analysis considered the relationship between AMA1 antibodies by ELISA and AMA1-specific inhibitory antibodies. AMA1-specific inhibitory antibodies were most prevalent for W2mef and FVO; therefore, these were the main focus of analyses. We found that high antibody responders (defined as greater than the median) to FVO and W2mef, determined by ELISA, had a higher prevalence of specific invasion inhibition of the *P. falciparum* line engineered to express the relevant AMA1 allele compared with low responders (*P* = 0.035 and 0.027, respectively, Fisher’s exact test), and a similar trend was observed for 3D7 (*P* = 0.16) but not Pf2006 (Fig. 6a). (Prevalence of specific inhibition for HB3 and XIE was too low to be included in this analysis.) However, many individuals with high levels of antibodies to an AMA1 allele (measured by ELISA) lacked evidence of AMA1-specific inhibitory antibodies. We also explored correlations between AMA1 allele-specific inhibitory activity and ELISA reactivity. Inhibitory antibodies were significantly more prevalent to the FVO allele than the 3D7 allele, or to the W2Mef allele compared to the XIE allele. However, there was no significant correlation between the relative inhibition of the FVO line versus the 3D7 expressing line and IgG reactivity to recombinant FVO AMA1 proteins by ELISA, but there was a significant correlation between the relative inhibition of W2Mef versus XIE and antibodies to W2Mef-AMA1 by ELISA (Spearman’s correlation ρ =0.55; *P* < 0.001). These findings suggest that ELISA data are indicative of functional activity to some extent, but they are not strongly or consistently predictive of AMA1-specific inhibitory activity and cannot be relied upon as a surrogate of inhibitory activity.

Total growth-inhibitory activity by serum antibodies against the lines expressing different AMA1 alleles was significantly correlated with levels of antibodies to the relevant AMA1 allele measured by ELISA (Spearman’s correlation ρ = 0.44–0.68, *P* < 0.0001; *n* = 98 or 99) (Additional file 1: Figure S5; Additional file 1: Table S2), suggesting that ELISA antibodies might broadly reflect the inhibitory potential of serum antibodies. However, note that the total invasion-inhibitory activity of antibodies in these assays may be contributed by antibodies to multiple antigens, not just AMA1, and antibody invasion-inhibitory activity against *P. falciparum* expressing a given AMA1 allele also correlated with IgG reactivity to heterologous AMA1 alleles in most instances, making it difficult to use standard ELISA as a surrogate for AMA1-specific invasion-inhibitory antibodies (Additional file 1: Table S2).

### Invasion inhibition versus allele-specific AMA1 antibodies measured by competition ELISA

We further investigated whether allele-specific antibodies measured by competition ELISA might be used as a surrogate of allele-specific, invasion-inhibitory antibodies. We measured allele-specific antibodies by competition ELISA amongst samples that showed at least 10 % greater inhibition of the W2-FVO AMA1 line than the W2-3D7 AMA1 line (*n* = 24) and amongst samples that did not show differential invasion inhibition (*n* = 64) (Fig. 6b). Amongst sera with FVO-specific invasion inhibition (indicating presence of invasion-inhibitory antibodies targeting FVO AMA1 but not 3D7 AMA1), there was a higher overall level of allele-specific antibodies to FVO AMA1 (that could not be competed by 3D7 AMA1 in competition ELISA) than allele-specific antibodies to 3D7 AMA1 (*P* < 0.0001) (Fig. 6b). However, many individuals with high levels of FVO AMA1-specific antibodies did not show significant FVO-specific invasion-inhibitory activity (Fig. 6b), suggesting that allele-specific antibodies measured by competition ELISA are not strong correlates of allele-specific inhibitory antibodies. Allele-specific, invasion-inhibitory antibodies probably form a subset of total allele-specific antibodies, suggesting that fine specificity, affinity or other antibody properties are important for inhibitory activity and that antibody levels measured by competition ELISA are not strongly predictive of functional activity.

### Prevalence of invasion-inhibitory AMA1 antibodies in Kenyan adults

**Fig. 7:**
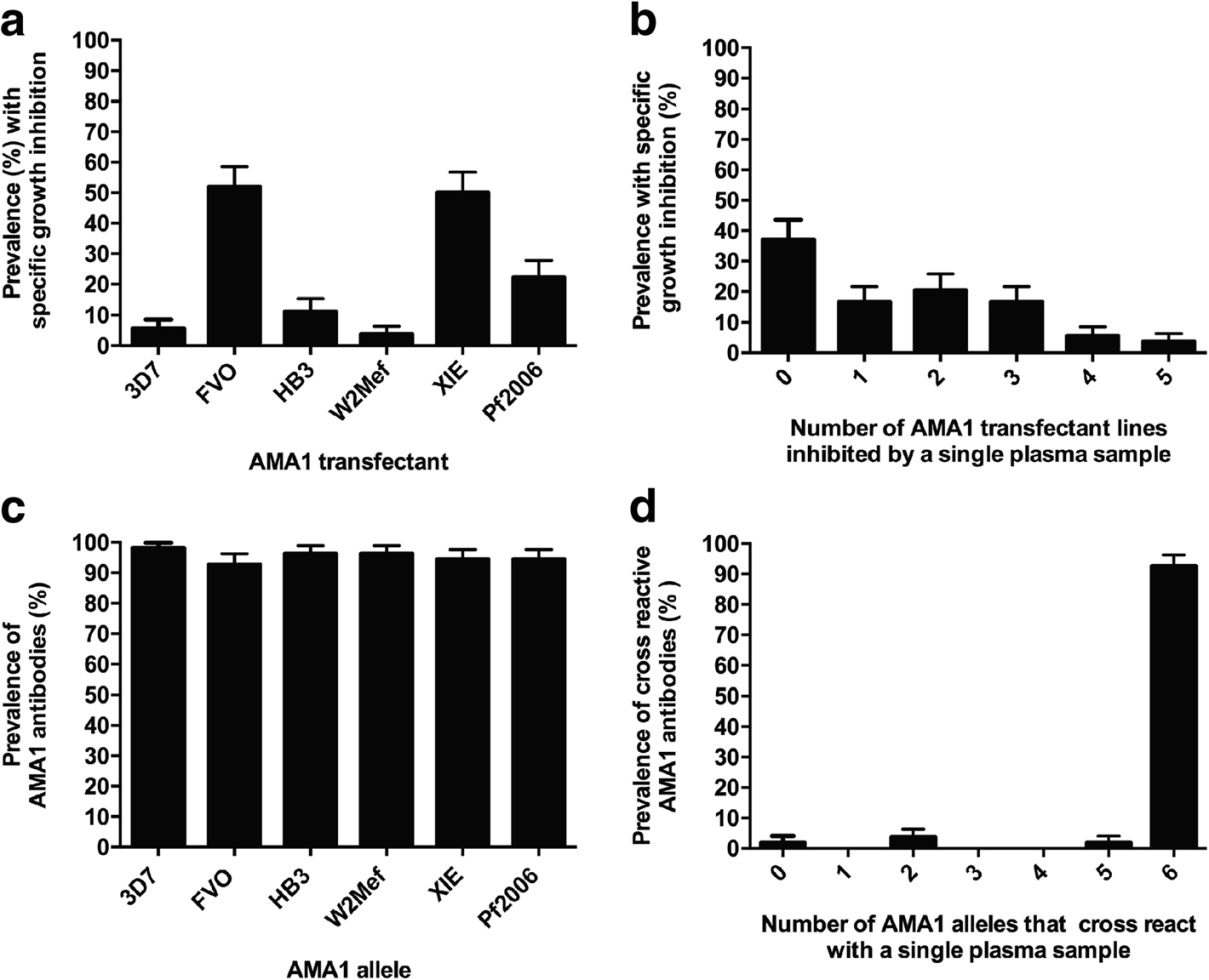
Prevalence of AMA1 allele-specific antibodies in Kenyan adults measured by invasion inhibition assays and ELISA. **a** Prevalence of AMA1 allele-specific invasion inhibition amongst adult Kenyan serum samples (*n* = 54). Error bars indicate standard error. **b** Prevalence of Kenyan serum samples capable of inhibiting the growth of more than one transgenic parasite line. Error bars indicate standard error. **c** Prevalence of antibodies to recombinant AMA1 by ELISA amongst Kenyan sera samples. Error bars indicate standard error. **d** Prevalence of Kenyan sera samples (*n* = 54) that were antibody-positive by ELISA for multiple AMA1 alleles

To investigate whether there are differences in the acquisition of AMA1 inhibitory antibodies between regions, we tested samples from adults in a malaria-endemic region of Kenya for invasion inhibition and antibodies to AMA1 by ELISA. The most prevalent inhibitory antibodies in Kenyan samples were to the FVO (52 %) and XIE (50 %) AMA1 alleles (Fig. 7a). This differs from PNG, where the highest prevalence was to the FVO and W2Mef AMA1 alleles, with a low prevalence of inhibitory antibodies to XIE. Inhibitory antibodies to Pf2006 were also more prevalent in Kenyan than in PNG samples. Furthermore, the levels of inhibitory antibodies were higher for FVO and XIE than the comparison lines, W2mef, HB3, Pf2006 or 3D7 (*P* < 0.0001; Wilcoxon’s matched-pairs signed-rank sum test). Analysis of the cross-reactivity of inhibitory antibodies revealed that most Kenyan serum samples were not broadly cross-inhibitory, with few samples inhibiting more than three different AMA1 lines (Fig. 7b), as seen with the PNG samples. A higher proportion of sera inhibited both FVO and XIE lines (79 % of FVO inhibitors inhibited XIE and 83 % of XIE inhibitors inhibited FVO) than was seen for the predominant inhibitory responses in PNG samples. Overall, results suggest there was broader inhibitory activity amongst Kenyan samples than PNG samples.

As was seen in PNG, the prevalence of AMA1 antibodies measured by ELISA was high (92–98 % classified as positive) and was similar for each of the six AMA1 alleles that were expressed by the genetically engineered *P. falciparum* lines (Fig. 7c). Antibody responses were strongly correlated between alleles (Spearman’s ρ = 0.86–0.95, *P* < 0.0001), and the majority of serum samples (92 %) were positive for each of the six AMA1 alleles tested (Fig. 7d) (relative antibody levels did not clearly reflect the pattern of inhibitory antibodies; Additional file 1: Figure S6). When Kenyan sera samples that showed an overlap in invasion-inhibitory activity were compared with those that inhibited only a single genetically engineered *P. falciparum* line (e.g. FVO and XIE versus FVO only), no difference was observed in antibody level by ELISA (*P* > 0.5184, Mann-Whitney U test) or absolute invasion inhibition (*P* > 0.3823). Hence, similar to the results found for PNG sera, the overlap in invasion-inhibitory activity of Kenyan sera is not related to antibody magnitude measured by ELISA. Total growth-inhibitory activity of lines expressing different AMA1 alleles by plasma antibodies was significantly correlated with levels of antibodies to the relevant AMA1 allele measured by ELISA (Additional file 1: Figure S7) (Spearman’s ρ = 0.51–0.68, *P* < 0.0001, *n* = 54; Additional file 1: Table S3). However, ELISA antibodies to heterologous AMA1 alleles were also significantly correlated with inhibition of a given isolate expressing a specific AMA1 allele (Spearman’s ρ = 0.44–0.69, *P* = 0.001 to *P* < 0.0001, *n* = 54; Additional file 1: Table S3). Therefore, it was difficult to use standard ELISA to quantify or estimate the presence of AMA1-specific inhibitory antibodies.

We further explored the relationship between AMA1 allele-specific inhibitory antibodies and the reactivity of antibodies to different AMA1 alleles measured by ELISA. We first evaluated the correlation between ELISA antibodies to FVO and 3D7 and the difference in inhibitory activity between *P. falciparum* isolates expressing FVO and 3D7 alleles (since inhibitory antibodies were highly prevalent to FVO, but not to 3D7). We found that FVO-specific inhibitory antibodies (relative to 3D7) were not correlated with antibodies to FVO by ELISA (Spearman’s ρ = –0.1101, *P* = 0.428). Further analysis by ELISA also found no correlation between antibodies to FVO and FVO-specific inhibitory antibodies relative to HB3 (Spearman’s ρ = –0.1706, *P* = 0.2173), W2Mef (Spearman’s ρ = –0.2281, *P* = 0.0972), XIE (Spearman’s ρ = –0.0845, *P* = 0.5435) or Pf2006 (Spearman’s ρ = –0.0633, *P* = 0.6488). Hence, while AMA1 antibodies by ELISA are significantly correlated with the total growth-inhibitory capacity of naturally exposed Kenyan sera (Additional file 1: Table S3), they do not appear to be predictive of AMA1-specific inhibitory activity.

## Discussion

The polymorphic nature of many malaria vaccine candidates presents major challenges to achieving highly efficacious vaccines. To date, there has been very little knowledge on the prevalence and patterns of functional immune responses to any polymorphic vaccine candidate in populations to guide vaccine design. Here we studied AMA1 as an important vaccine candidate and as a model for understanding functional immunity to polymorphic vaccine antigens in populations. We successfully developed a novel approach using *P. falciparum* engineered to express different polymorphic variants of AMA1 to quantify AMA1 as a target of naturally acquired inhibitory antibodies, thereby determining their prevalence, strain specificity and functional importance in immune sera. Our results reveal that AMA1 is a major target of invasion-inhibitory human antibodies, and these antibodies have a strong strain-specific component to their activity. A highly significant finding is that the prevalence of inhibitory antibodies varied substantially for different alleles, and this prevalence differed between geographic regions. This has major implications for the selection of alleles for inclusion in a multi-allele vaccine. Our studies showed that inhibitory antibodies to the FVO allele were highly prevalent in both geographic regions, and inhibitory antibodies were also prevalent to W2Mef and XIE alleles/serotypes, identifying them as strong candidates for inclusion in future AMA1 vaccines. These findings have major implications for vaccine design, and it has not previously been possible to detect such serotypic differences in functional immune responses by standard immunoassays. Moreover, for the first time, our approach enables measurement of inhibitory antibodies at a population level, which can be used to guide the selection of AMA1 alleles for inclusion in a future multi-allele vaccine. To our knowledge, the allele/serotype-specific prevalence of functional antibodies in populations has not been reported for any malaria antigen or vaccine candidate to date. These functional immunoassays will also be highly valuable for evaluating immune responses and monitoring vaccine escape in vaccine trials.

The selection of specific alleles, strains or serotypes for inclusion in multi-allele or multi-strain vaccines typically needs to consider the predominant alleles or strains circulating in target populations. This approach has been used extensively with vaccines for other pathogens, including influenza, pneumococcus, meningococcus and human papilloma virus. We propose that the prevalence of allele-specific inhibitory antibodies in target populations should be considered in vaccine design, and this study develops new tools to measure this. Our studies establish an approach that could be used to evaluate functional antibodies for other vaccine candidates and quantify the importance of allele specificity and population prevalence of allele-specific antibodies. Given the enormous investment cost in taking vaccine candidates through pre-clinical studies, GMP production and into clinical trials, ensuring vaccine design addresses issues of antigenic diversity and vaccine escape is essential.

Data from multi-allele competition ELISAs amongst human populations and studies of vaccine-induced antibodies in rabbits suggest that the diversity in AMA1 may be covered by only a small number of different AMA1 alleles [31, 32, 42, 43]. Tailoring vaccine design to include the most prevalent alleles/serotypes, based on assays measuring functional antibodies, would further facilitate allele selection to maximize population coverage by AMA1 vaccines. In this study, the higher prevalence of inhibitory antibodies to FVO and W2mef alleles in the PNG population and FVO and XIE in the Kenyan population almost certainly reflects greater exposure to these alleles in the respective study populations. Our findings support the potential inclusion of these three alleles (or closely related alleles) in a multi-allele AMA1 vaccine, particularly FVO, which was a prominent target of inhibitory antibodies in both populations. However, data are needed from other populations to further inform these selections and better define the extent of regional differences. As there are more than 200 recorded unique alleles of AMA1 [12–15, 30–32] and no evidence of geographic clustering [33, 34], it is unlikely that identical forms of any one of the six AMA1 alleles tested in this study are circulating at a high prevalence in either population examined. Instead, it is likely that there are a number of antigenically similar variants circulating, leading to the acquisition of functional antibodies. As such, each of the AMA1 alleles included in this study appears to represent a member of a distinct serotype or serogroup of AMA1 alleles that each share a collective set of epitopes targeted by invasion-inhibitory antibodies.

Analysis of AMA1 sequences suggested that the ability of AMA1 alleles to recruit T cell help is not a differentiating factor in the ability of each AMA1 allele to elicit functional antibody responses. Whilst the number of human major histocompatibility complex (MHC) class II binding peptides predicted to be present in AMA1 varies greatly depending on the human leukocyte antigen (HLA) allele examined, there is no significant difference in the number of MHC class II binding peptides for a given HLA allele across each of the six AMA1 alleles examined in this study (data not shown; NetMHCII 2.2 server: http://www.cbs.dtu.dk/services/NetMHCII/). There are also no data to suggest that the FVO, W2mef or XIE alleles are inherently more susceptible to inhibitory antibodies, and studies have shown comparable inhibition of different alleles by vaccine-induced antibodies [42, 43].

This is the first human population study to quantify AMA1 as a target of naturally acquired invasion-inhibitory antibodies and estimate the prevalence of these inhibitory antibodies. Both children and adults in PNG showed similar prevalence of AMA1-specific invasion inhibition, and adults had a tendency for a higher prevalence of inhibitory antibodies. In contrast to AMA1, inhibitory antibodies to merozoite surface protein 1 (C-terminal region) were lower in both populations [39, 44].

Acquired human inhibitory AMA1 antibodies have substantial allele-specific activity, rather than cross-reactive inhibition. The strain-specific nature of human inhibitory antibodies we observed is consistent with the strain-specific efficacy reported in a phase II AMA1 vaccine trial [2]. There was limited overlap in the inhibitory activity of antibodies to different alleles. Whilst a high prevalence of antibodies to all AMA1 alleles was found by ELISA in PNG and Kenyan samples, invasion-inhibitory antibodies to the FVO and W2mef AMA1 alleles in PNG and FVO and XIE AMA1 alleles in Kenya were much more prevalent than invasion-inhibitory antibodies to other AMA1 alleles. We explored the relationship between antibodies to AMA1 measured by standard ELISA, or allele-specific antibodies measured by competition ELISA, and AMA1-specific inhibitory antibodies. We did find some significant associations between antibodies measured by ELISA and AMA1-specific inhibitory antibodies for some AMA1 alleles. However, these associations were not consistent for all alleles, and overall, ELISA values were not highly predictive of allele-specific inhibitory antibodies. This highlights the potential limitations of relying on standard immunoassays that do not assess functional activity, which is influenced by antibody fine specificity, avidity and other factors that are not measured by ELISA. For example, a previous study reported that the growth-inhibitory activity of naturally acquired antibodies did not significantly correlate with AMA1 antibodies measured by standard ELISA; however, measuring antibodies to an inhibitory epitope of AMA1 showed a better correlation with growth inhibition [28]. Furthermore, AMA1 vaccine trials in malaria-exposed populations have reported that the induction of AMA1 antibodies measured by ELISA does not necessarily predict the induction of functional inhibitory antibodies [45, 46]. It has also been reported that some acquired antibodies may interfere with the activity of AMA1-specific invasion-inhibitory antibodies [29]. We did find significant correlations between AMA1 antibody reactivity and total growth-inhibitory activity of serum antibodies. However, total growth-inhibitory activity is mediated by antibodies to multiple antigens that are co-acquired with increasing exposure to malaria [47], not just by antibodies to AMA1. These observations and considerations further emphasise the need for functional assays to evaluate AMA1 in populations and clinical trials.

Our data are consistent with malaria-exposed individuals acquiring a repertoire of allele-specific invasion-inhibitory antibodies of multiple specificities, rather than cross-inhibitory antibodies. Although ELISA data indicate that humans acquire some cross-reactive antibodies [12, 20, 21, 32], it might be that polymorphisms have concentrated around functional epitopes in order to facilitate evasion of protective antibodies [21]. The most polymorphic residues in AMA1 occur adjacent to the hydrophobic trough, which acts as a binding site for RON2 [10, 48, 49]. Malaria exposure leads to the acquisition of antibodies to a highly polymorphic inhibitory epitope around this region [28]. A longitudinal study showed a strong association between polymorphisms  in AMA1 and the development of symptomatic malaria and infection episodes [50]. Previous studies have shown that rabbit antibodies raised against different AMA1 alleles inhibit invasion of a panel of *P. falciparum* isolates to varying degrees, but may also show some cross-inhibitory activity depending on the alleles being tested [31]. The present data suggest that human antibodies are more strongly allele-specific than antibodies from immunised animals; this highlights the importance of studying human antibody responses to inform vaccine development and the caveats in extrapolating results from vaccine studies performed in small laboratory animals to human populations. Although our ability to detect any samples with broadly cross-inhibitory antibodies to AMA1 could potentially be reduced using our approach, this appears unlikely to be a major issue because our data, and those of others [32], suggest that broadly cross-reactive antibodies to AMA1 do not occur, or are rare, amongst humans; furthermore, they are not generated by standard immunisation approaches [31, 42]. It is not possible to delete the AMA1 gene and use AMA1-knockout parasites in functional assays, because AMA1 is essential in *P. falciparum*; therefore, allele replacement was the only approach possible to quantify antibodies in functional assays. The predominantly allele-specific nature of AMA1 inhibitory antibodies demonstrated by these data indicates that a multi-allele vaccine approach, or alternate strain-covering approach, will be required for AMA1.

## Conclusions

We successfully developed and applied a novel approach to quantify functional immune responses to a polymorphic vaccine candidate in malaria-exposed populations using genetically engineered parasites. Our findings indicate that AMA1 is a key target of invasion-inhibitory antibodies that are largely allele-specific, and that the prevalence of antibodies to different alleles varies substantially within and between populations. These findings indicate that vaccine design, such as the selection of specific alleles for inclusion in a multi-allele vaccine, may need to take account of the different population prevalence of functional antibody serotypes. These findings are broadly relevant to understanding immunity and vaccine design for the many candidate malaria vaccine antigens that have similar issues of antigen diversity, and suggest that further studies are urgently needed to determine the distribution of different serotypes or antigenic variants of leading vaccine antigens, and the patterns of functional immunity to these variants in different populations, to guide rationale vaccine development and achieve maximal efficacy.

## Additional file

Additional file 1: Detailed methods and additional analysis of antibody data. (DOCX 1576 kb)

## Abbreviations

AMA1: Apical membrane antigen 1
ELISA: Enzyme-linked immunosorbent assay
GIA: Growth inhibition assay
HLA: Human leukocyte antigen
PNG: Papua New Guinea
