# Supplementary material for: A novel approach to identifying patterns of human invasion-inhibitory antibodies guides the design of malaria vaccines incorporating polymorphic antigens
Source: BMC Med. 2016 Sep 23;14:144. doi: 10.1186/s12916-016-0691-6 (PMC5034621; doi:10.1186/s12916-016-0691-6)
Supplement: Additional file 1: — Detailed methods and additional analysis of antibody data. (DOCX 1576 kb) [file 12916_2016_691_MOESM1_ESM.docx]

**Additional file 1**

**Methods**

***Generation of genetically engineered W2Mef P.falciparum expressing HB3, XIE or Pf2006 AMA1 alleles.***

The DNA sequences encoding HB3, XIE and Pf2006 AMA1 alleles were codon optimised for expression in *E. coli* (Genecript). AMA1 alleles were inserted into the plasmid vector pCC1AMA1TP.1 and transfected into W2Mef strain of parental parasites as previously described [[1](#_ENREF_1)]. Parasite populations confirmed to have plasmid integration into the AMA1 target after positive selection with WR99210 were cloned by limiting dilution, and positive populations confirmed by Southern blot. AMA1 alleles 3D7, FVO, W2Mef, and HB3 were selected because they have been previously shown to be antigenically different [[1](#_ENREF_1)]. We included XIE and Pf2006 as examples of recent PNG and African isolates [[1](#_ENREF_1)].

***SDS-PAGE (Polyacrylamide gel electrophoresis) and Immunoblot analysis***

SDS-PAGE and immunoblot analysis of parasite proteins was performed as previously described [[1](#_ENREF_1)]. Synchronised schizont-stage parasite cultures were saponin-lysed, washed in phosphate buffered saline (PBS) and resuspended in non-reducing SDS sample buffer (Invitrogen). Samples were sonicated and heated to 100^0^C for 5 minutes prior to SDS-PAGE. Proteins were separated on 3-8% Tris-Acetate gels (Invitrogen) and transferred onto nitrocellulose using the iBlot system (Invitrogen) according to standard protocols. Blots were probed with the relevant anti-AMA1 rabbit antiserum (1:2000 sera), or purified IgG (20μg/ml IgG). Blots were also probed with a mouse monoclonal antibody generated against the *P. falciparum* HSP-70 protein (1:2000) as a loading control. Horseradish peroxidase-coupled (HRP) goat anti-rabbit Ig (1:2000) or sheep anti-mouse Ig (1:2000) Millipore) were used as secondary antibodies.

***P. falciparum invasion inhibition assays***

Flow cytometry based invasion inhibition assays were performed as described in detail elsewhere [[1](#_ENREF_1)]. Synchronized pigmented trophozoite stage parasites were cultured with 1/10 diluted heat inactivated PNG, Kenyan and non-malaria exposed Melbourne control human sera samples for 72 hours. After two invasion cycles, early trophozoite stage parasites were stained, and parasitemia measured by flow cytometry. Percent growth inhibition = (% parasitemia in test well/mean % parasitemia in non-inhibitory control sera/ IgG/ RPMI/ DMF/PBS) x100) -100) x-1. All PNG samples were tested in duplicate or triplicate in at least 2 independent assays and the mean of the results were used. All Kenyan samples were tested in duplicate in a single assay, and the mean of the results used. Parasite lines expressing different AMA1 alleles were tested in parallel for inhibition by serum samples. At least 3 different lines were tested in each assay, and results were pooled from all assays for analysis. A subset of 66 samples were tested against all 6 parasite lines in parallel to further validate the findings.

***ELISAs and competition ELISAs***

Standard ELISAs to measure AMA1 antibodies in plasma were performed as described [[2](#_ENREF_2)]. All AMA1 proteins were diluted to a concentration of 1.0 μg/ml in PBS and coated onto Maxisorp microtiter plates (Nunc) (100μl/well, overnight at 4^0^C). Duplicate dilutions of human samples were incubated for 2 hours. Secondary antibody was polyclonal sheep anti-human IgG HRP Ab (Millipore) used at 1/2500 and incubated for 1 hour. All wash steps were done in PBS/0.05% Tween 20. All blocking and antibody dilutions were in 5% skim milk/PBS/0.05% Tween 20, with incubations at room temperature. Color was developed with an ABTS liquid substrate system (Sigma) and stopped after 15 min with 1% NaDodSO_4_ (SDS). OD was measured at 405 nm using a GENios microplate reader (Tecan).  Background absorbance was determined using PBS controls on each plate and was deducted from all other values.  For competition ELISAs, serum samples (1:1000 dilution) were mixed with competing AMA1 antigen (5ug/ml) and allowed to pre-incubate at room temperature for 30 minutes before adding to the AMA1 coated plate and continuing as for standard ELISAs [[3](#_ENREF_3)].

**Supplementary Figures**

**
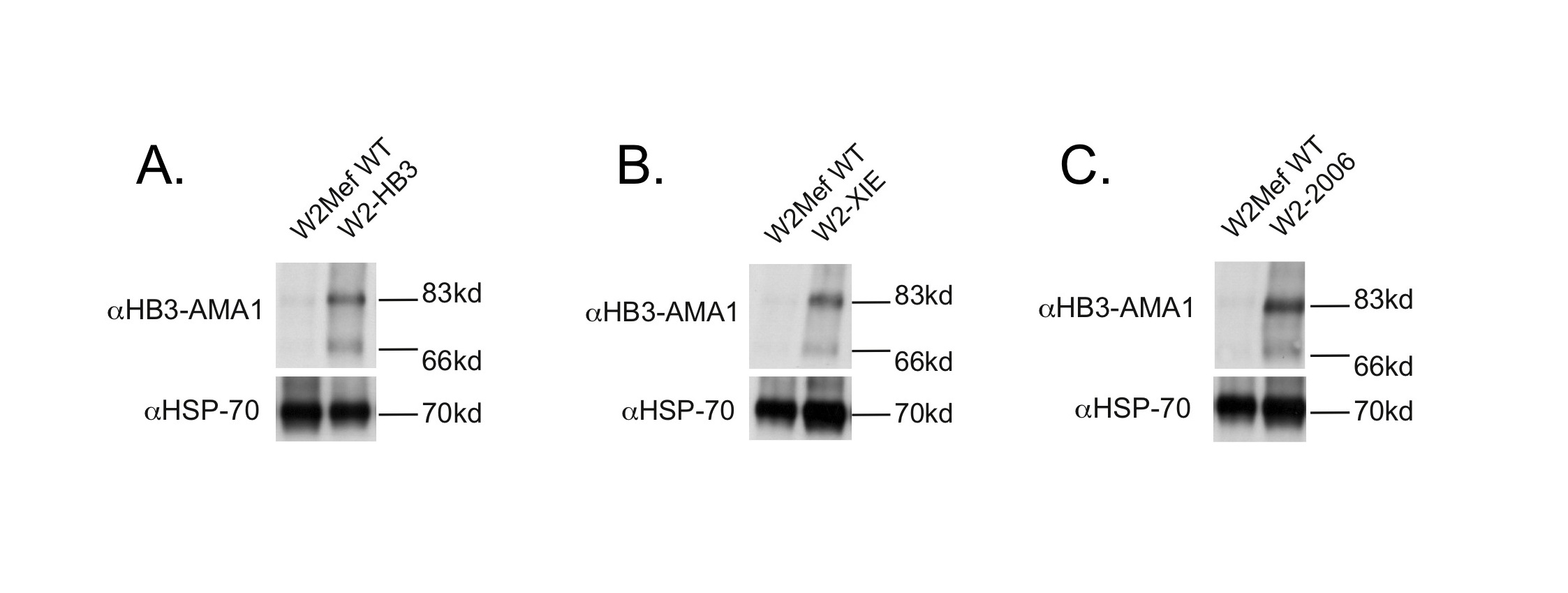
**

**Figure S1.** **Phenotypic analysis of genetically engineered *P.falciparum* lines.** **(A)** W2-HB3, **(B)** W2-XIE, **(C)** W2-2006. Proteins extracted from synchronised schizont-stage parasites were probed by Western blot with rabbit antibodies raised against HB3 AMA1 (known to cross-react with XIE and Pf2006, but not W2mef). Blots were also probed with a mouse monoclonal antibody raised against HSP-70 as a loading control.


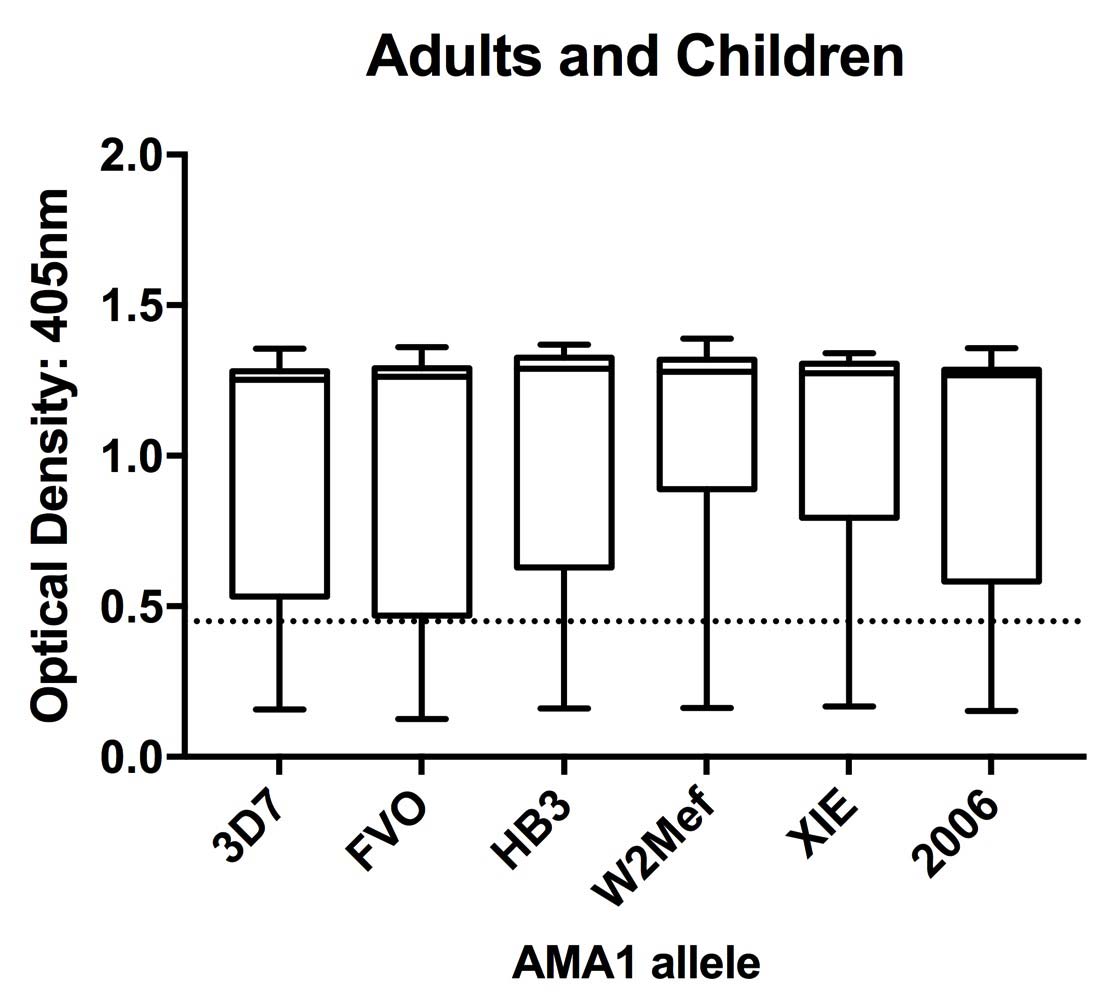


**Figure S2. Comparison of antibody levels in PNG sera against different AMA1 alleles**. Comparison of antibody levels measured against six AMA1 alleles among samples from PNG children and adults tested together in the same experiment. The median, interquartile range, minimum and maximum optical density measured at 405nm are shown (n=13 Adult and n=23 children)


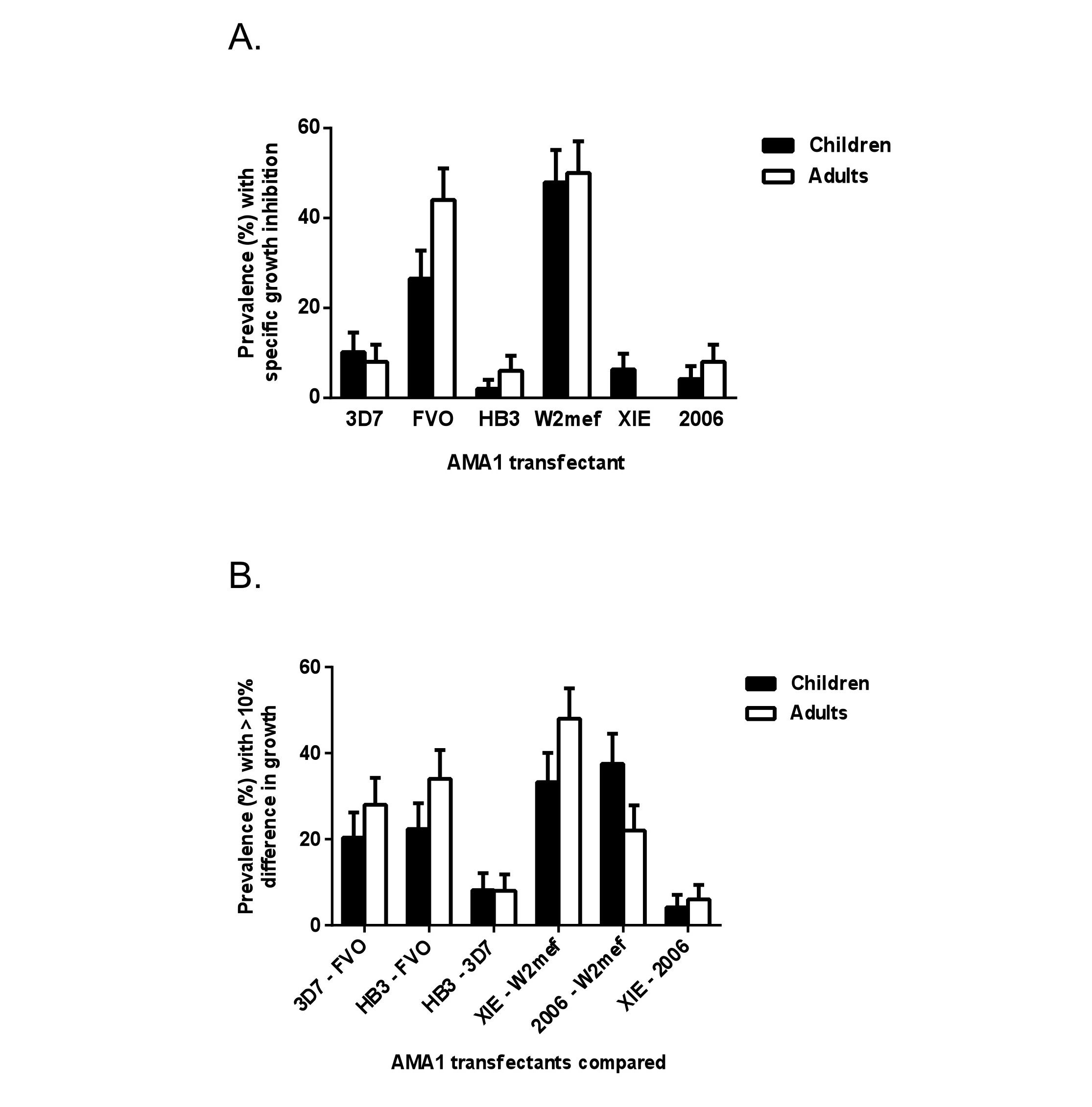


**Figure S3. Differential invasion inhibition of genetically engineered *P.falciparum* expressing alternate AMA1 alleles**. **(A)** Prevalence of specific invasion inhibition (at least 10% greater inhibition of one genetically engineered *P.falciparum* line compared with either of two other lines tested at the same time) amongst PNG serum samples from children (n= 48 or 49) and adults (n = 50). **(B)** Prevalence of PNG serum samples showing difference in growth >10% between two lines (line A – line B) tested simultaneously amongst children (n= 48 or 49) and adults (n = 50). Comparisons were made between lines tested together in the same assays (3D7, FVO and HB3, or W2mef, XIE and Pf2006). Error bars indicate +SEM.


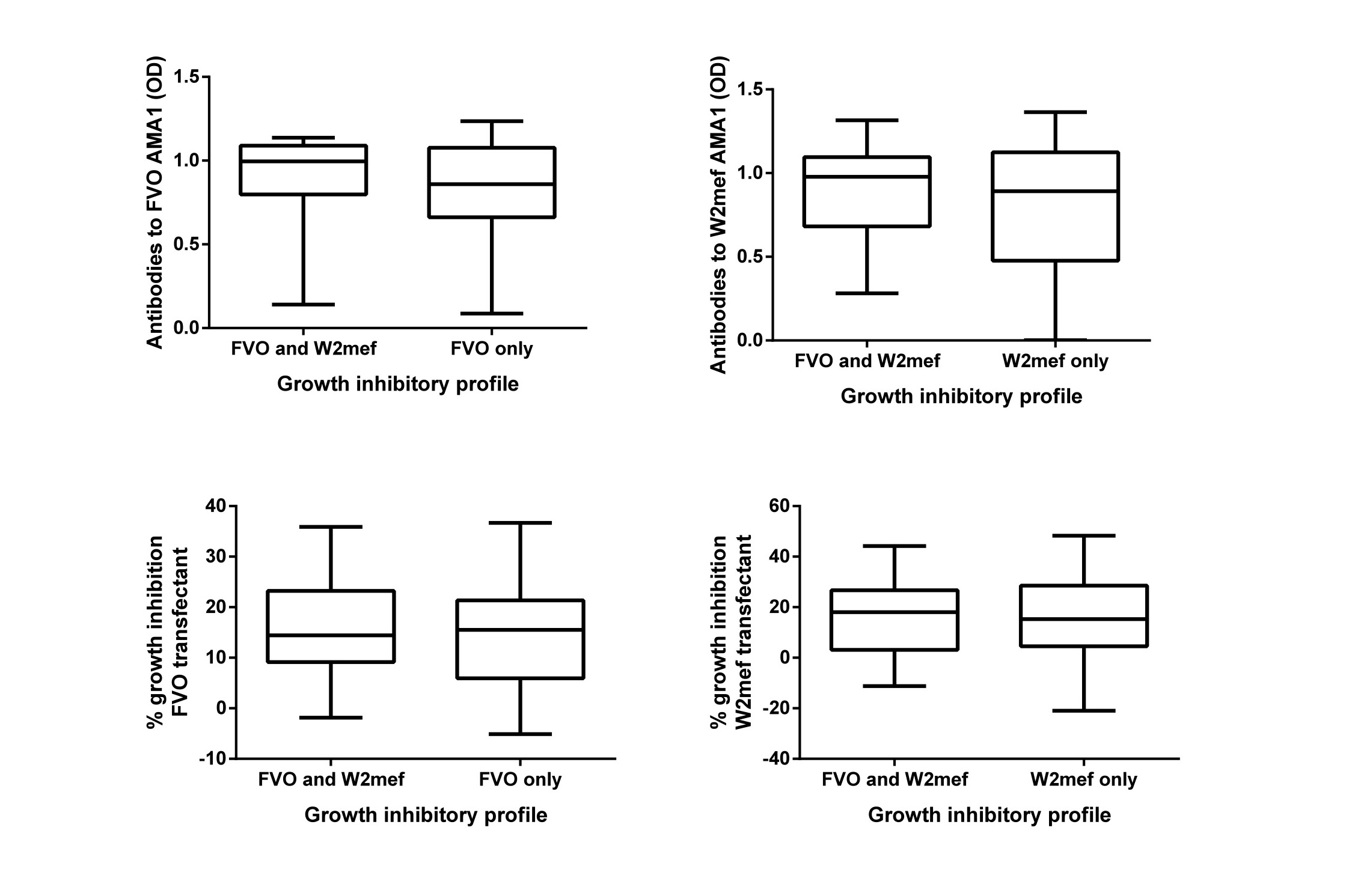


**Figure S4. Antibody levels and growth inhibitory activity in samples with inhibitory activity against two lines compared with samples that inhibited only one line.**

**
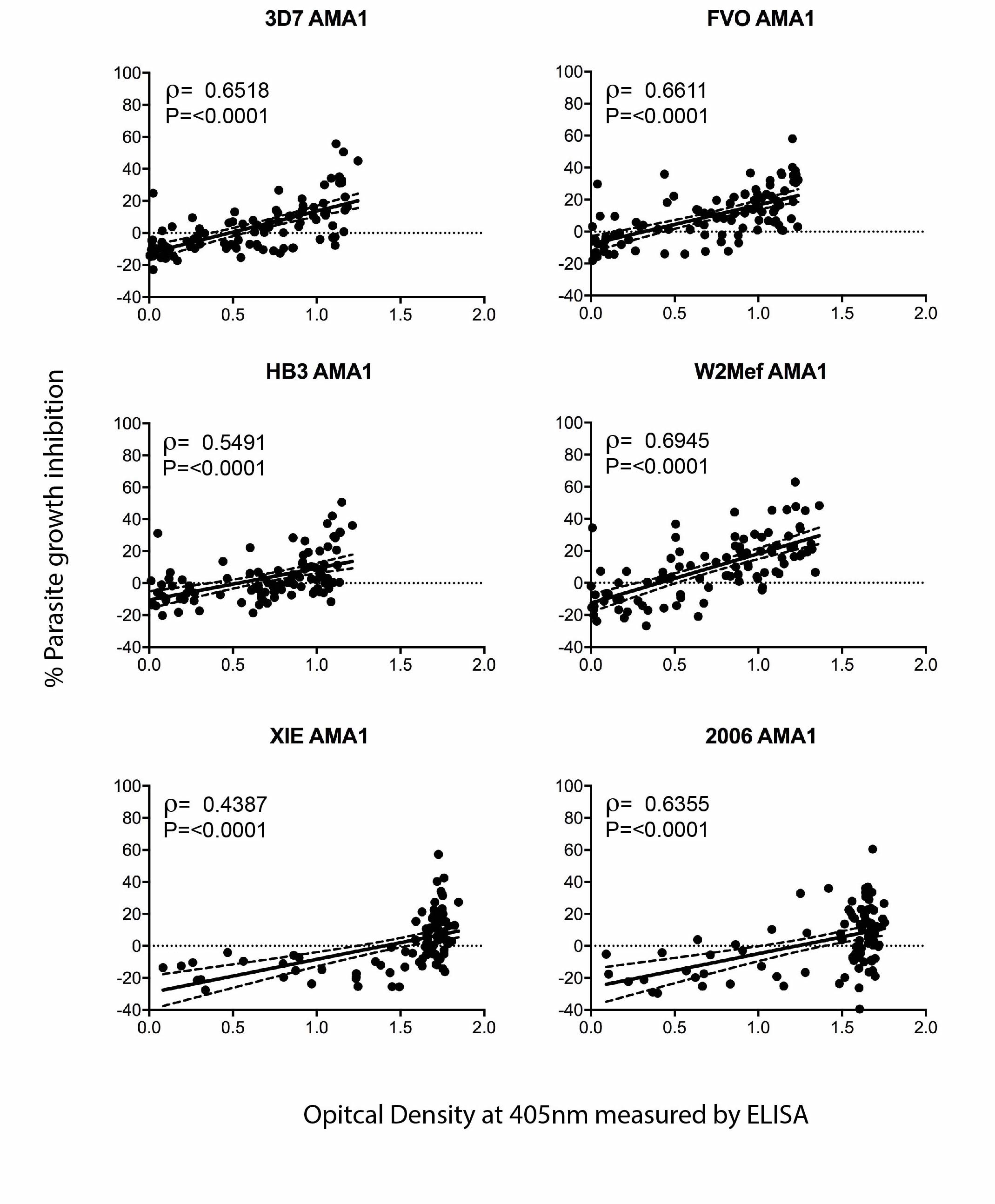
**

**Figure S5. Antibody levels and growth inhibitory activity correlations for PNG samples.** The relationships between antibody levels measured by ELISA and total growth inhibition for each AMA1 allele are shown in scatter plots. The inhibition data used for each comparison was the total growth inhibition of the *P. falciparum* line expressing the same AMA1 allele as used in ELISA. N=98 or 99.


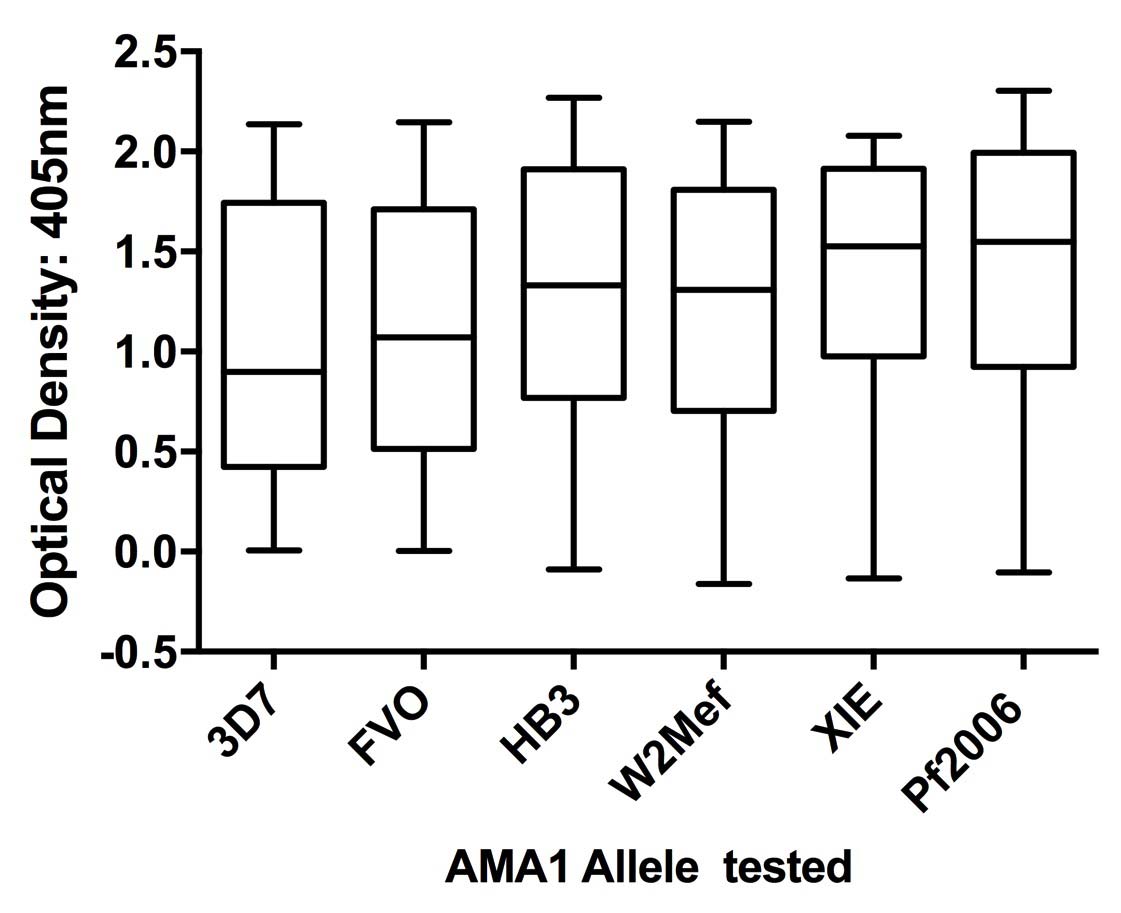


**Figure S6. Antibody levels among Kenyan sera against different AMA1 alleles**. Antibody levels measured against six AMA1 alleles by ELISA; all samples were tested against all alleles in the same assay. The median, interquartile range, minimum and maximum optical density measured at 405nm for N=54 Kenyan sera samples.


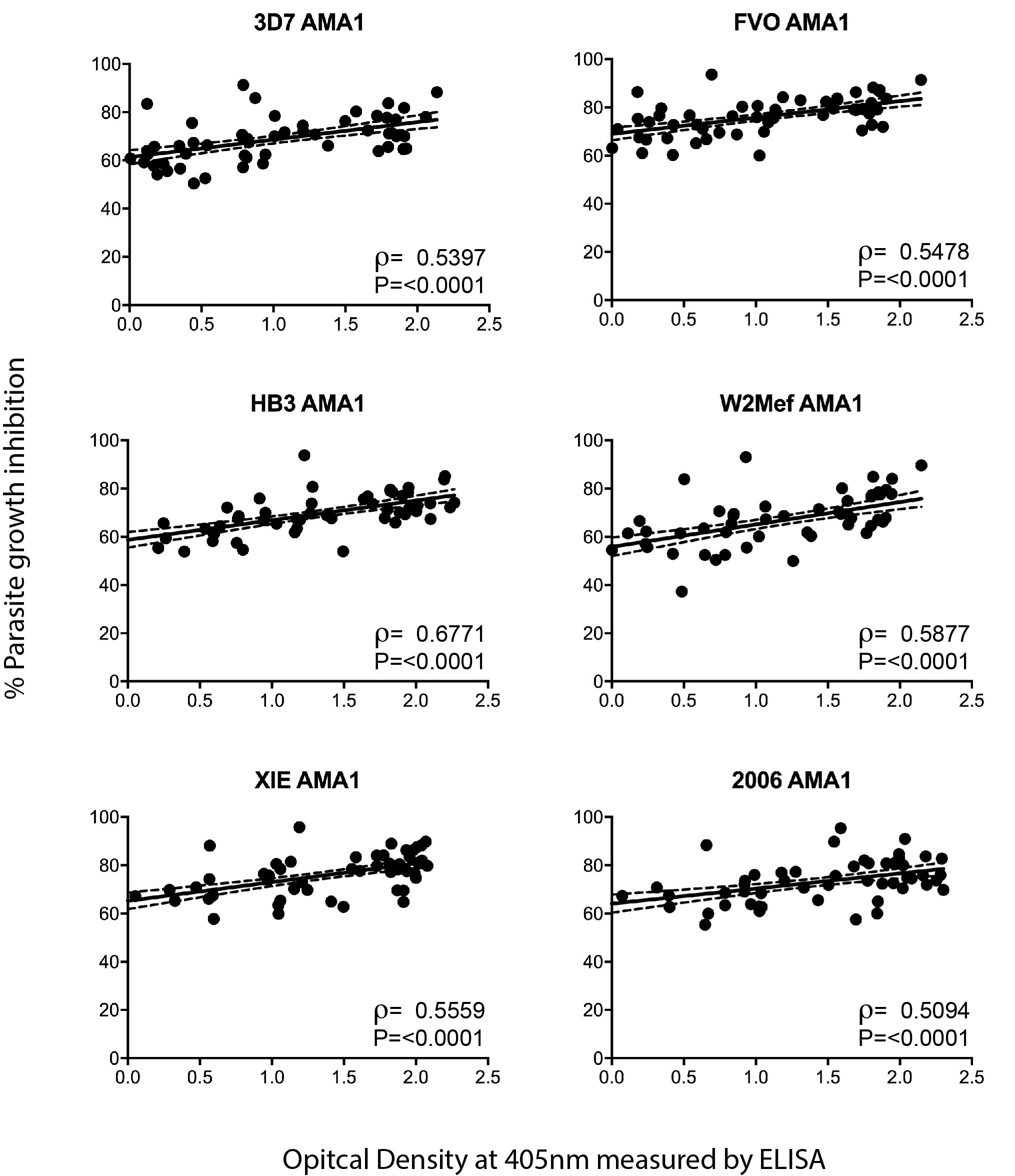


**Figure S7. Antibody levels and growth inhibitory activity correlations for Kenyan samples.** The associations between antibody levels measured by ELISA and growth inhibition measured by GIA for each AMA1 allele are shown. The data used for each comparison was the total growth inhibition of the *P. falciparum* line expressing the same AMA1 allele as used in ELISA. N=54.

**Table S1.** Profile of specific inhibition of AMA1 transfectants by PNG sera (n = 99).

| **Number of AMA1 transfectants inhibited by a single serum sample** | **AMA1 alleles** | **Number of sera** |
| --- | --- | --- |
| 1 | FVO | 13 |
|  | W2mef | 23 |
|  | 3D7 | 1 |
|  | HB3 | 0 |
|  | Pf2006 | 1 |
|  | XIE | 1 |
| 2 | FVO and W2mef | 13 |
|  | FVO and 3D7 | 3 |
|  | FVO and HB3 | 1 |
|  | FVO and Pf2006 | 1 |
|  | W2mef and 3D7 | 4 |
|  | W2mef and XIE | 2 |
|  | W2mef and Pf2006 | 2 |
| 3 | FVO, W2mef and HB3 | 2 |
| 4 | FVO, W2mef, 3D7 and Pf2006 | 1 |
|  | FVO, W2mef, HB3 and Pf2006 | 1 |

Allele-specific inhibition defined as inhibition where one AMA1 transfectant line was inhibited at least 10% more than either of two other lines tested in parallel (3D7, FVO and HB3; W2mef, XIE and Pf2006)

**Table S2:** Correlation between antibodies to AMA1 alleles by ELISA and total growth inhibitory activity for samples from PNG donors

|  | **ELISA Values** | | | | | |
| --- | --- | --- | --- | --- | --- | --- |
|  | **3D7** | **FV0** | **HB3** | **W2mef** | **Pf2006** | **XIE** |
| **3D7 inhibition** | ρ = 0.6518  P =<0.0001 | ρ = 0.5970  P =<0.0001 | ρ = 0.6244  P =<0.0001 | ρ = 0.2210  P = 0.0342 | ρ = 0.0355  P = 0.7369 | ρ =-0.0506  P = 0.6315 |
| **FV0 inhibition** | ρ = 0.6463  P =<0.0001 | ρ = 0.6611  P =<0.0001 | ρ = 0.6538  P =<0.0001 | ρ = 0.2351  P = 0.0241 | ρ = 0.0472  P = 0.6546 | ρ =-0.0481  P = 0.6488 |
| **HB3 inhibition** | ρ = 0.5252  P =<0.0001 | ρ = 0.5311  P =<0.0001 | ρ = 0.5491  P =<0.0001 | ρ = 0.1118  P = 0.2886 | ρ = 0.0790  P = 0.4541 | ρ =-0.0005  P = 0.9959 |
| **W2mef inhibition** | ρ = 0.3317  P = 0.0012 | ρ = 0.2784  P = 0.0055 | ρ = 0.3049  P = 0.0031 | ρ = 0.6945  P =<0.0001 | ρ = 0.1270  P = 0.2276 | ρ = 0.6732  P = 0.5238 |
| **Pf2006 inhibition** | ρ =-0.0909  P = 0.3887 | ρ =-0.0547  P = 0.6044 | ρ =-0.0817  P = 0.4384 | ρ = 0.09523  P = 0.3665 | ρ = 0.4387  P =<0.0001 | ρ = 0.5428  P =<0.0001 |
| **XIE inhibition** | ρ =-0.1479  P = 0.1594 | ρ =-0.1101  P = 0.2943 | ρ =-0.1332  P = 0.2056 | ρ = 0.0299  P = 0.7766 | ρ = 0.5292  P =<0.0001 | ρ = 0.6355  P =<0.0001 |

ρ= Spearman’s rank-order correlation coefficient (rho) for the correlation between antibodies to recombinant AMA1 measured by ELISA and total invasion inhibitory activity (not AMA1-specific inhibition) against *P. falciparum* lines expressing different AMA1 alleles

Shaded boxes indicate a significant correlation (p<0.05)

**Table S3:** Correlation between antibodies to AMA1 alleles by ELISA and total growth inhibitory activity for antibodies from Kenyan donors

|  | **ELISA Values** | | | | | |
| --- | --- | --- | --- | --- | --- | --- |
|  | **3D7** | **FV0** | **HB3** | **W2mef** | **Pf2006** | **XIE** |
| **3D7 inhibition** | ρ = 0.5397  P =<0.0001 | ρ = 0.5342  P =<0.0001 | ρ = 0.5490  P =<0.0001 | ρ = 0.5790  P =<0.0001 | ρ = 0.5564  P =<0.0001 | ρ = 0.5484  P =<0.0001 |
| **FVO inhibition** | ρ = 0.5343  P =<0.0001 | ρ = 0.5478  P =<0.0001 | ρ = 0.5938  P =<0.0001 | ρ = 0.5857  P =<0.0001 | ρ = 0.6019  P =<0.0001 | ρ = 0.5789  P =<0.0001 |
| **HB3 inhibition** | ρ = 0.6549  P =<0.0001 | ρ = 0.6564  P =<0.0001 | ρ = 0.6771  P =<0.0001 | ρ = 0.6608  P =<0.0001 | ρ = 0.6871  P =<0.0001 | ρ = 0.6661  P =<0.0001 |
| **W2mef inhibition** | ρ = 0.5192  P =<0.0001 | ρ = 0.5489  P =<0.0001 | ρ = 0.5041  P =<0.0001 | ρ = 0.5877  P =<0.0001 | ρ = 0.4786  P = 0.0003 | ρ = 0.5004  P = 0.0003 |
| **Pf2006 inhibition** | ρ = 0.4378  P = 0.0010 | ρ = 0.4671  P = 0.0003 | ρ = 0.4843  P = 0.0002 | ρ = 0.5058  P =<0.0001 | ρ = 0.5094  P =<0.0001 | ρ = 0.4865  P = 0.0002 |
| **XIE inhibition** | ρ = 0.5041  P = 0.0001 | ρ = 0.5231  P =<0.0001 | ρ = 0.5789  P =<0.0001 | ρ = 0.5289  P =<0.0001 | ρ = 0.5852  P =<0.0001 | ρ = 0.5559  P =<0.0001 |

ρ= Spearmans rank-order correlation coefficient (rho) for the correlation between antibodies to recombinant AMA1 measured by ELISA and total invasion inhibitory activity (not AMA1-specific inhibition) against *P. falciparum* lines expressing different AMA1 alleles

References

1. Drew DR, Hodder AN, Wilson DW, Foley M, Mueller I, Siba PM, Dent AE, Cowman AF, Beeson JG: **Defining the antigenic diversity of Plasmodium falciparum apical membrane antigen 1 and the requirements for a multi-allele vaccine against malaria**. *PLoS One* 2012, **7**(12):e51023.

2. Stanisic DI, Richards JS, McCallum FJ, Michon P, King CL, Schoepflin S, Gilson PR, Murphy VJ, Anders RF, Mueller I *et al*: **Immunoglobulin G subclass-specific responses against Plasmodium falciparum merozoite antigens are associated with control of parasitemia and protection from symptomatic illness**. *Infect Immun* 2009, **77**(3):1165-1174.

3. Terheggen U, Drew DR, Hodder AN, Cross NJ, Mugyenyi CK, Barry AE, Anders RF, Dutta S, Osier FH, Elliott SR *et al*: **Limited antigenic diversity of Plasmodium falciparum apical membrane antigen 1 supports the development of effective multi-allele vaccines**. *BMC Med* 2014, **12**(1):183.
